# Digital Cognitive Assessment Tests for Older Adults: Systematic Literature Review

**DOI:** 10.2196/47487

**Published:** 2023-12-08

**Authors:** Claudio Cubillos, Antonio Rienzo

**Affiliations:** 1 Escuela de Ingeniería Informática Pontificia Universidad Católica de Valparaíso Valparaíso Chile; 2 Escuela de Ingeniería Biomédica Universidad de Valparaiso Valparaíso Chile

**Keywords:** cognitive digital test, systematic review, cognitive screening, digital interventions, older adults

## Abstract

**Background:**

The global health pandemic has affected the increasing older adult population, especially those with mental illnesses. It is necessary to prevent cases of cognitive impairment in adults early on, and this requires the support of information and communication technologies for evaluating and training cognitive functions. This can be achieved through computer applications designed for cognitive assessment.

**Objective:**

In this review, we aimed to assess the state of the art of the current platforms and digital test applications for cognitive evaluation, with a focus on older adults.

**Methods:**

A systematic literature search was conducted on 3 databases (Web of Science, PubMed, and Scopus) to retrieve recent articles on the applications of digital tests for cognitive assessment and analyze them based on the methodology used. Four research questions were considered. Through the PRISMA (Preferred Reporting Items for Systematic Reviews and Meta-Analyses) methodology, following the application of inclusion and exclusion criteria, a total of 20 articles were finally reviewed.

**Results:**

Some gaps and trends were identified regarding the types of digital applications and technologies used, the evaluated effects on cognitive domains, and the psychometric parameters and personal characteristics considered for validation.

**Conclusions:**

Computerized tests (similar to paper-and-pencil tests) and test batteries (on computers, tablets, or web platforms) were the predominant types of assessments. Initial studies with simulators, virtual environments, and daily-life activity games were also conducted. Diverse validation methods and psychometric properties were observed; however, there was a lack of evaluations that involved specific populations with diverse education levels, cultures, and degrees of technology acceptance. In addition, these evaluations should consider emotional and usability aspects.

## Introduction

### Background

According to the United Nations, a country is considered old when ≥7% of its population is aged >60 years [1,2]. However, some countries exceed this percentage [3-5]. The increase in life expectancy and the growing population of older adults represent some of the most significant demographic changes that society is experiencing today. In all countries, individuals aged ≥60 years are beginning to constitute a large segment of the population. It is estimated that the proportion of this population group will increase 2-fold, rising from 11% to 22% by the year 2050 [6]. Although there are significant variations between countries and continents, the segment of the population aged 60 years is the fastest growing [7]. In addition, 10.4% of adults aged >60 years have cognitive impairments associated with aging. In this group, 20.9% of people aged >80 years have this condition [8]. This affects the social and economic aspects of a country [9,10].

However, it is not the process of aging itself that is causing alarm among current governments and societies, but rather the burden of dementia that is associated with this aging population. The probability of developing dementia increases with age, doubling approximately every 5 years. In general, it is estimated that there are approximately 40 million people with dementia worldwide, with approximately 9 million people in Europe alone. Furthermore, this global figure is estimated to double every 2 decades, reaching 131.5 million by 2050 [11,12].

Due to the aforementioned reasons, there has been an increasing interest in cognitive training and other interventions that can mitigate or reverse these degenerative changes in older adults. There are several research papers and recent literature on the outcomes of cognitive interventions for brain training in older adults [12-15], but their scientific positions vary, and all authors emphasize the need for more empirical evidence. Furthermore, cognitive decline and changes in cognitive status [16] can easily go unnoticed in clinical settings. Cognitive assessments are often time-consuming and require a trained health care specialist, such as a neurologist or gerontologist [17,18], to provide detailed information on the patient’s health [19]. However, conducting these evaluations poses significant access barriers, as many older adults are unable to attend promptly due to physical or cognitive limitations, fear of going out (due to the pandemic), long waiting times, or long travel distances. These barriers constitute important obstacles in determining the initial stages of cognitive decline.

Thus, this study aimed to provide an updated literature review of the main specialized digital cognitive tests for older adults. This review classified the tests based on their types and characteristics. In addition, it included comparative tables that highlight the technological aspects, cognitive domains evaluated, tasks, activities, and psychometric parameters used in each test.

### Cognitive Problems in Older Adults

**Figure 1:**
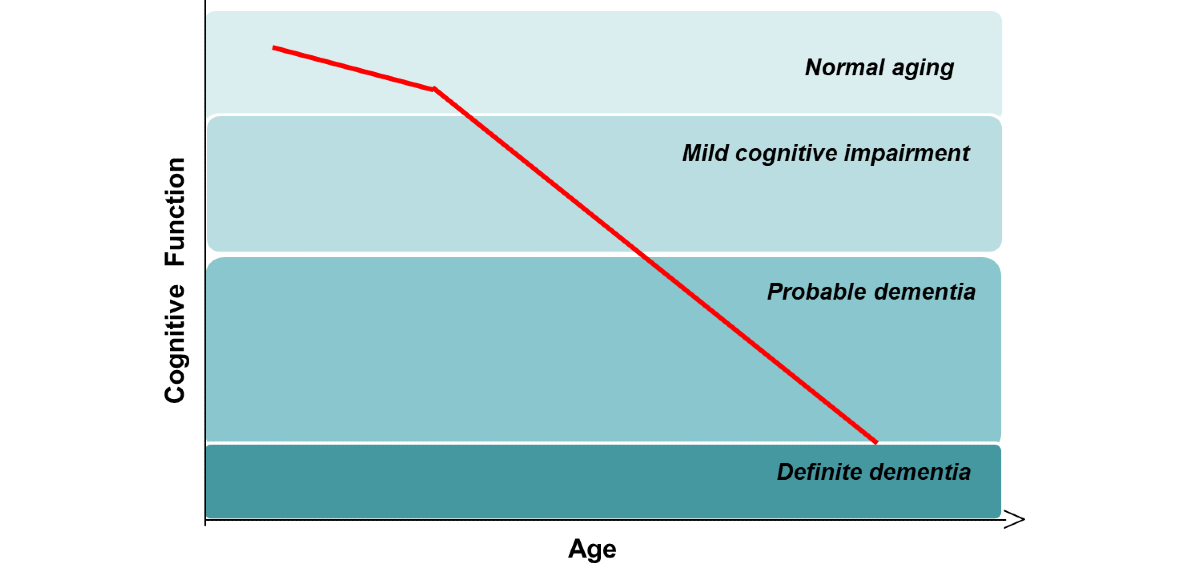
Changes in cognitive functions in a person.

As a normal component of aging, many people experience a decline in their cognitive functions. When the decline becomes more significant, pathological processes may occur. Different levels of cognitive impairment were observed. As cognitive degeneration progresses, cognitive and functional decline reach a threshold, and the person is clinically diagnosed with probable dementia [20]. At present, it is unlikely that neuronal damage in the brain can be reversed, and most of the recent treatments available only provide symptom relief rather than a cure for the disease. However, the disease progression can be effectively controlled if dementia is detected at an early stage. Therefore, the most effective strategy is to detect dementia in its early stages and initiate an intervention. Theoretical changes in cognitive function in a person as a function of age (toward possible dementia) are shown in Figure 1 [20].

In recent years, the concept of mild cognitive impairment (MCI) has been applied to describe the transitional stage between normal aging and the early stages of dementia [21]. It refers to a “mild” condition in which there is objective memory impairment without functional impairment [21,22]. In general, individuals with MCI have a high probability of gradually progressing to dementia. This means that they are at a higher risk of developing dementia than healthy individuals. Approximately 12% to 15% of individuals with MCI develop clinical dementia with functional disability within 1 year; therefore, the early detection of dementia depends on an accurate diagnosis [21,22].

According to specialists [1], there are various types of cognitive impairment. Currently, amnesic and nonamnesic MCI are distinguished. This distinction is based on the presence or absence of deterioration in the mnemic function. In addition, it is possible to differentiate MCI according to the number of affected cognitive domains. Some individuals have unidomain MCI, whereas others have multidomain MCI, which involves impairment in >1 cognitive domain. Although memory impairment is the most representative symptom of MCI, several cognitive domains other than memory are compromised in most individuals with MCI [20,23,24].

### Theoretical Background

**Figure 2:**
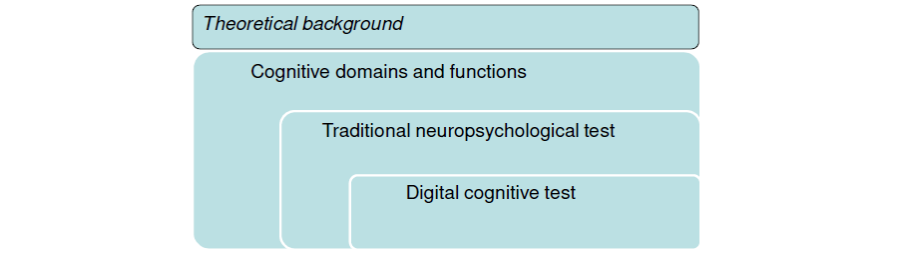
Structure of the theoretical background.

A summary is presented on the theoretical background of cognition and cognitive functions, and then it delves into the traditional evaluation tests as well as the digital cognitive tests that are available. The scheme of the work is shown in Figure 2.

### Cognitive Domains and Functions

For specialists, cognition is defined as “the set of psychological skills that account for all mental life,” and it is composed of “cognitive domains” (sets of cognitive processes or functions). Cognitive functions are mental processes that allow us to perform any task. They enable an individual to actively participate in the processes of receiving, selecting, transforming, processing, storing, and retrieving information, thus enabling them to function effectively in their surroundings. Cognitive skills are continuously used to learn and remember information, integrate personal history and identity, manage information related to the individual’s location and destination, maintain and distribute attention, recognize different sounds, process different stimuli, perform calculations, and mentally represent an object [25,26].

According to the literature, the most important cognitive functions include attention [25,27,28], orientation [27,29], memory [25,27], perception (or gnosis) [25,26,29], executive functions [26,29], praxis [25,26,29], language [25,29], social cognition [26,29], and visuospatial skills [26,29]. Cognitive disorders affect the cognitive functions of individuals who experience them.

A special area of interest (for older adults) turns out to be memory, which deserves further explanation. It is the most frequently mentioned cognitive function, the lack of which is attributed to making most daily errors. Apparently, everything is attributed to the “lack” of memory, which is difficult to define. However, in general terms, memory refers to the ability to acquire, store, and retrieve various types of information [25,30]. At each stage of the memory process, the entire brain is involved [27], encompassing several phases: registration, encoding, storage, recall, and recognition of information. There are different forms and types of memory [25,27], including sensory or iconic memory; short-term memory (STM); working memory (also called operational memory); and long-term memory, which can be divided into 2 groups [29-31]: explicit or declarative memory and implicit or nondeclarative memory.

### Traditional Neuropsychological Tests

Neuropsychological evaluation is used to determine an individual’s cognitive status. It is conducted when there are symptoms of cognitive syndromes such as aphasia or dementia. The evaluation consists of performing cognitive tests to establish the presence of these syndromes. For example, if it is necessary to determine a patient’s language proficiency, a cognitive test assessing language skills should be conducted. Similarly, if it is necessary to determine a patient’s memory status, a cognitive test evaluating their memory should be performed. Therefore, neuropsychological evaluation can determine the presence of cognitive or behavioral syndromes and suggest the etiology of the pathological condition as well as the possible evolution. This knowledge is crucial for determining appropriate rehabilitation measures for patients [32].

Several cognitive assessment techniques have been developed to assess adults in clinical settings. They can be distinguished between tests for cognitive screening, which diagnose possible levels of cognitive impairment or degrees of dementia, and specialized tests that evaluate one or more specific cognitive domains [16,33]. A previous literature review established that the most common screening types are the Montreal Cognitive Assessment (MoCA) [34,35], the Mini-Mental State Examination (MMSE) [30,36,37], the Addenbrooke’s Cognitive Examination-Revised [31], the Mini-Cog [35] and the Abbreviated Mental Test (AMT) [37,38]. Among the tests used to evaluate cognitive abilities in specific domains and in patients with special characteristics are the following: the Stroop test, which is an attentional test that detects neurological and brain problems and assesses the ability to classify information from the environment and react selectively to it [39]; the Corsi Cubes test, which evaluates visuospatial STM and allows the study of the effect of emotions on STM [40]; the Trail Making Test (TMT), a neuropsychological test that measures visual attention and task switching (consisting of 2 parts) [41]; and the Rey-Osterrieth Complex Figure test, which provides information about a person’s neuropsychological functioning [42] in terms of attention, concentration, coordination, and visuospatial abilities, among others.

Most cognitive tests are evaluated by specialists by using psychometric parameters. Psychometry is a branch of experimental psychology that is responsible for measuring and quantifying a person’s psychological processes and cognitive abilities [43]. The most commonly used measures to assess the quality of psychometric instruments, as well as the measurement parameters that are normally used in cognitive tests, include reliability [44], validity [45], sensitivity [44,46], specificity [44,46], and receiver operating characteristic (ROC) curves [47]. These measures are briefly explained in Multimedia Appendix 1.

### Digital Cognitive Tests

New computing technologies and platforms, including tablets and smartphones, offer many opportunities to create interactive tasks and experiences that can be used to infer the cognitive status. Cognitive assessment software packages are available [48,49], and they offer computerized versions of traditional tests that can be self-administered. In addition, various websites perform tests and training for certain cognitive abilities. Examples include Cognifit [50], NeuronUP [51], and Stimulus [52].

An analysis of previous literature reviews yielded 4 papers by the following authors: Zygouris and Tsolaki [53] in 2015, Aslam et al [54] in 2017, Marques-Costa et al [55] in 2018, and Tsoy et al [56] in 2021. These reviews included 11 to 17 papers that were published in 2012, 2015, 2017, and 2019, respectively. The most recent study by Tsoy et al [56] focused on only 3 cognitive domains: attention, memory, and language. From the above, it can be deduced that there is a need for an up-to-date state-of-the-art technology. Previous reviews have mainly focused on computer tests and digital test batteries, neglecting the inclusion of emerging technologies such as virtual reality (VR), video games, gamification, and artificial intelligence (AI). Tsoy et al [56] used this as an exclusion criterion for his systematic review.

Previous reviews provide different levels of detail regarding the characteristics of different digital tests. These include the hardware used [53,54,56], input mode or data capture [53,54], test time [53-56], and administration modality [53,54]. However, none of them offer details on the instructions and how they are delivered to the patient, the environment or place where the test is performed, or the sequence of tasks that the participants must perform. This lack of information makes it difficult to compare the results within the same cognitive domain.

One aspect analyzed by all these reviews was the quality of the cognitive tests and the psychometric properties applied. They agree on highlighting important drawbacks in terms of the replicability of the studies [54], existence of well-structured psychometric data [55], and evaluation of various psychometric properties [53]. However, a limitation of these reviews is that they do not include the sample size in the tests, details of how many participants had MCI or dementia, or psychometric parameters used for each test in their comparative tables. All these aspects are addressed in this review.

The main specialized digital cognitive tests for older adults are presented, classifying and comparing the domains and cognitive tasks evaluated. This complements the missing characteristics in the analyses of the previous reviews.

## Methods

### Objectives and Research Questions

To investigate the current state of applications or digital tests for cognitive evaluation in older adults, 4 research questions were formulated:

- RQ1: What are the different technological alternatives that are currently used in digital devices to assess the cognitive abilities of older adults?
- RQ2: What are the different types and characteristics of computerized (digital) cognitive tests?
- RQ3: What are the main characteristics of the subtests and tasks used in different digitized cognitive tests?
- RQ4: What are the main effects, personal traits, and psychometric parameters considered for validating digitized cognitive tests for older adults?

### Eligibility Criteria

A literature review was conducted on digital systems for detecting cognitive problems. The aim was to gather updated information on technological solutions that could help overcome the possible barriers and difficulties of traditional psychometric tests. To do this, the PRISMA (Preferred Reporting Items for Systematic Reviews and Meta-Analyses) methodology was used. Through a standardized review process, information is delivered in a flowchart that considers 4 stages: identification, screening, eligibility, and inclusion.

To search primary studies (articles), we searched 3 databases based on the search strategies: Web of Science, PubMed, and Scopus. These databases were chosen because they have peer review processes in which experts approve the publications. We combined the keywords with logical operators to obtain the following search expression:

(“cognitive assessment” OR neuropsych*) AND (computer* OR web OR “digital test” OR evaluation) AND (“older adult” OR adult) AND (“cognitive impairment”) AND valid*.

Due to the high number of publications (although a large percentage of articles appear on several sites), the following criteria were considered for the first selection and review of the most relevant articles:

- Inclusion criteria were articles and research papers that were published 2015 onward and were written in both English and Spanish; those articles in which titles and summaries (abstracts) included terms that addressed any of the research questions. The articles should be published by publishers with a website and should fall under the categories of scientific articles, conferences (proceedings), or book chapters.
- Exclusion criteria were articles in which titles and abstracts were not related to the objective of the study or the research questions, those that were repeated in another language, and those that were not related to older adults.

### Data Collection

**Figure 3:**
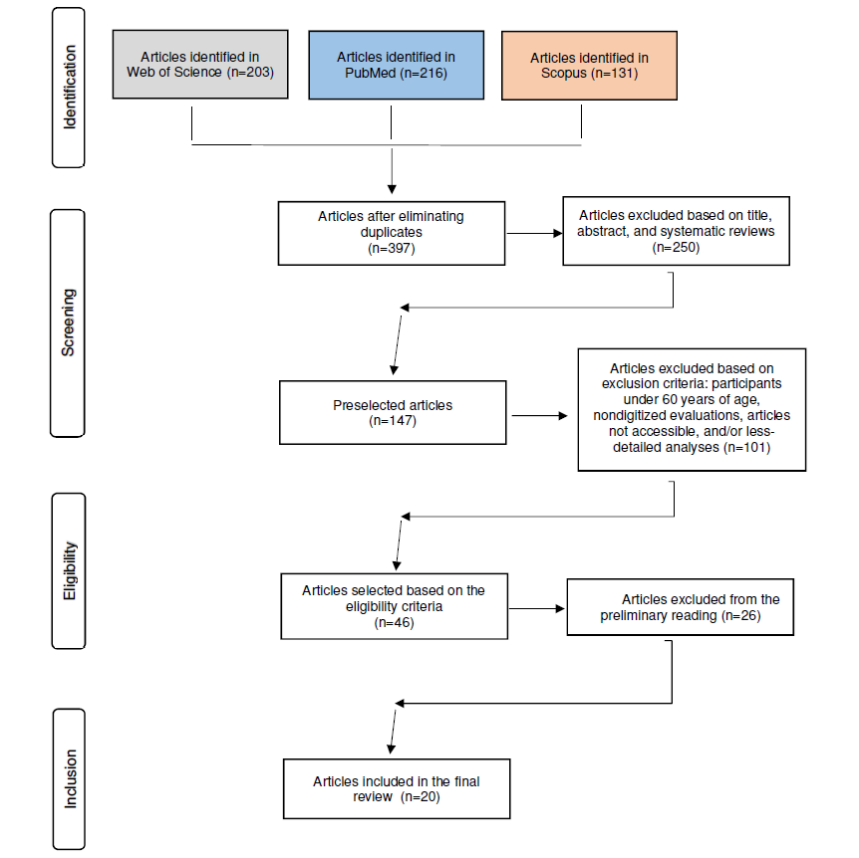
PRISMA (Preferred Reporting Items for Systematic Reviews and Meta-Analyses) scheme of the systematic review (own elaboration).

On the basis of the proposed methodology, we searched for articles from the 3 databases. The process is shown in the flowchart in Figure 3. The explanation for each phase of the PRISMA methodology is as follows:

### Identification

A systematic search of the literature was performed using 3 detailed databases, resulting in the retrieval of 203 articles from Web of Science, 216 articles from PubMed, and 151 articles from Scopus.

- Screening: duplicates were eliminated, leaving 397 articles. The searches were then filtered by title, abstract, and systematic review, resulting in a total of 147 reports.
- Eligibility: after conducting the first superficial reading of the articles (n=101), those that did not meet the inclusion criteria were excluded, resulting in 46 studies.
- Inclusion: these 46 documents were reviewed again to identify points that did not align with the study objectives. Finally, 20 articles were selected. All articles corresponded to scientific articles; there were no conference proceedings, book chapters, or university theses.

In a previous search, various validated methodologies were found concerning the so-called “gold standard,” or reference test. These tools allow for different methodologies to be used in conducting structured tests to quantify the affected cognitive domain. Table 1 was created using 20 articles discovered. Each article included the first author’s name, year of publication, country, instrument name, and the technological alternative that met certain quality criteria.

**Table 1:** Articles analyzed.

Study, year	Country	Article title	Types of technology
Köstering et al [57], 2016	Germany	Analyses of rule breaks and errors during planning in computerized tower tasks: insights from neurological patients	Computerized cognitive tests
Scharre et al [58], 2017	United States	Digitally translated Self-Administered Gerocognitive Examination (eSAGE): Relationship with its validated paper version, neuropsychological evaluations, and clinical assessments	Digital version
Wong et al [59], 2017	China	Computerized Cognitive Screen: a self-administered computerized test for screening for cognitive impairment in community social centers	Computerized cognitive battery
Valladares-Rodriguez et al [60], 2017	Spain	Design process and preliminary psychometric study of a video game to detect cognitive impairment in senior adults	Computerized cognitive game
Rapp et al [61], 2018	United States	Computer simulations for assessing cognitively intensive instrumental activities of daily living in older adults	Simulator of daily activity
Possin et al [62], 2018	United States	The Brain Health Assessment for detecting and diagnosing neurocognitive disorders	Cognitive web platform
Groppell et al [63], 2019	United States	A rapid, Mobile Neurocognitive Screening Test to aid in identifying cognitive impairment and dementia (BrainCheck): cohort study	Cognitive web platform
Khaligh-Razavi et al [64], 2019	United States	Integrated Cognitive Assessment: speed and accuracy of visual processing as a reliable proxy to cognitive performance	Computerized cognitive tests
Eraslan Boz et al [65], 2019	Turkey	A new tool to assess amnestic mild cognitive impairment in Turkish older adults: Virtual Supermarket	Virtual reality environment
Takahashi et al [66], 2019	Japan	Development and validity of the Computer-Based Cognitive Assessment Tool for intervention in community-dwelling older individuals	Computerized cognitive battery
Ichii et al [67], 2019	Japan	CogEvo, a cognitive function balancer, is a sensitive and easy psychiatric test battery for age-related cognitive decline	Computerized cognitive battery
Schulz-Heik et al [68], 2020	United States	Evaluation of adding the CANTAB^a^ computerized neuropsychological assessment battery to a traditional battery in a tertiary care center for veterans	Computerized cognitive battery
Cahn-Hidalgo et al [69], 2020	United States	Validity, reliability, and psychometric properties of a computerized, cognitive assessment test (Cognivue)	Computerized cognitive battery
Tsoy et al [70], 2020	United States	BHA-CS^b^: A novel cognitive composite for Alzheimer’s disease and related disorders	Cognitive web platform
Chin et al [71], 2020	Korea	A validation study of the Inbrain CST^c^: a tablet computer-based Cognitive Screening Test for Elderly People with cognitive impairment	Computerized cognitive battery
Lunardini et al [72], 2020	Italy	Supervised digital neuropsychological tests for cognitive decline in older adults: usability and clinical validity study	Computerized cognitive tests
Noguchi-Shinohara et al [73], 2020	Japan	A new computerized assessment battery for cognition (C-ABC) to detect mild cognitive impairment and dementia around 5 min	Cognitive web platform
Chan et al [74], 2020	China	Electronic cognitive screen technology for screening older adults with dementia and mild cognitive impairment in a community setting: development and validation study	Cognitive web platform
Rodriguez-Salgado et al [75], 2021	Cuba	A brief digital cognitive assessment for detection of cognitive impairment in Cuban older adults	Cognitive web platform
Bottiroli et al [76], 2021	Italy	The smart aging platform for assessing early phases of cognitive impairment in patients with neurodegenerative diseases	Computerized cognitive game

^a^CANTAB: Cambridge Neuropsychological Test Automated Battery.

^b^BHA-CS: Brain Health Assessment-Cognitive Score.

^c^CST: Cognitive Screening Test.

## Results

### Overview

Some preliminary statistics could be obtained regarding the publication dates of the articles, such as the countries where the studies were conducted (Table 1). Subsequently, the technological alternatives were classified and explained. For example, differentiating between digital tests, that is, if they are cognitive batteries and computerized cognitive tests, or computerized platforms (web), or simulators of daily activity, using a VR environment, or computerized cognitive games. Possible factors that influenced each psychometric instrument were also characterized. For example, the technical aspects of the tests such as the administration time, data capture mode, modality (technology used), operation (instructions) of the software, and how the tests are administered.

In addition, comparative tables were created to provide a synthesized description of the digitized cognitive tests, differentiating some characteristics such as the cognitive domains covered, the number of tests or stages available, the activities involved in each test, and the evaluation scoring system. Finally, the articles were analyzed to validate their psychometric properties in comparison with traditional tests.

Statistical data of the articles studied are shown in Tables 2 (based on the year of publication) and 3 (based on the country where the study was conducted).

**Table 2:** Papers by the year of publication.

Year of publication	Papers (n=20), n (%)
2016	1 (5)
2017	3 (15)
2018	2 (10)
2019	5 (25)
2020	7 (35)
2021	2 (10)

**Table 3:** Papers by the country of study.

Countries for study	Papers (n=20), n (%)
United States	8 (40)
Europe	5 (25)
Japan	3 (15)
Chinese	2 (10)
Korea	1 (5)
Cuba	1 (5)

### RQ1: What Are the Different Technological Alternatives That Are Used in Digital Devices to Assess the Cognitive Abilities of Older Adults?

#### Overview

To answer the first research question (RQ1), we conducted a preliminary analysis of the selected articles, identified differences in categories of digital tests, and briefly described the identified classes. Table 4 shows the number of articles on each application (according to the type of technology).

**Table 4:** Papers by the type of digital test (technology).

Types of digital test (technology)	Papers (n=20), n (%)
Test battery	6 (30)
Web platform	6 (30)
Computerized test	3 (15)
Games	2 (10)
Digital version	1 (5)
Simulator IADL^a^	1 (5)
Virtual reality	1 (5)

^a^IADL: Independent Activities of Daily Living.

#### Cognitive Batteries and Computerized Cognitive Tests

These are a set of tests and tasks that allow the evaluation of multiple cognitive domains, such as language, executive function, attention, and memory [57,59]. The tests are based on and validated using psychometric methods. They present a greater advantage than traditional tests (pencil and paper), as they lead to a detailed cognitive profile, reducing possible errors caused by administration bias [59]. In addition, they provide information about the testing process, such as reaction times and the sequence of answers (good and not good). In addition, automatic scoring helps professionals improve clinical diagnoses. The small difference between test batteries [59,66-69,71] and computerized tests [57,64,72] is that the former has several tests and tasks designed to assess >4 different cognitive domains, whereas the latter includes fewer tests and focuses on a single cognitive domain. In general, computerized cognitive batteries, which are used on PCs, notebooks, or tablets, serve as supportive tools in clinical and community settings.

#### Simulators of Daily Activity

At the onset of MCI, the ability to perform daily activities remains unaffected. However, as the deterioration progresses, the performance of these activities decreases. Activities such as shopping, taking medications, and using telephone lines become difficult. To assess the condition, it is necessary to invest in devices and time and to consider the burden it places on the patients. As an alternative, the use of simulators for daily activities offers enormous possibilities. For example, in Simulation-Based Assessment of Cognition [61], the patient can interact with software to complete tasks such as withdrawing money at a virtual automated teller machine or making a call to a virtual pharmacy to indicate the needed medicine. These activities are associated with executive function. Therefore, it is an instrument with many advantages that require further development to include the full range of activities commonly performed by older adults, vary the performance depending on the particular neuropathology, and adapt to different cultural and socioeconomic settings.

#### Computerized Platforms (Web)

These are cognitive batteries, tasks, or adaptations of traditional tests that allow comprehensive evaluations of cognitive impairment using mobile devices or devices connected to the internet. The cognitive evaluation begins after patients enter their sociodemographic data. Among these options, the platforms can be configured with an algorithm that enables the questions to be displayed and prompts the participant to select the correct answer. Meanwhile, the information is stored on a centralized computer server, which allows access to cognitive detection to be faster, more efficient, and automatic; and it is possible to perform the test in the comfort of one’s home (or any appropriate place) [62,63,70,73-75].

#### Digital Version

These are digital cognitive assessments that are equivalent to paper versions and allow for greater flexibility. In addition, the tool (a single test, not a battery) can increase the screening of individuals who are being evaluated through self-administration using technological devices. The technological solution is built based on the questions from the original version but with the added advantage of automatically measuring the time it takes for participants to answer the questions. In addition, it can determine the frequency with which participants return to previous pages based on the subject. Finally, the evaluation is accessible through web, and the results obtained are delivered in a digital format [58].

#### VR Environment

Currently, there is an increase in the use of VR technology for evaluating cognitive dysfunction. An individual can enhance their interaction in a simulated environment by following the instructions of a traditional cognitive test. For example, in a Virtual Supermarket (VSM) [65], before the exercise, age, gender, occupation, years of education, and any possible memory complaints are registered. VSM generates a randomized list of products for a daily shopping activity. The individual is expected to locate the items on the list, place them in the shopping cart, take them to the register and pay the correct amount. Furthermore, the participant must navigate the VSM by touching green footprints on the screen while pushing the shopping cart. It is an exercise designed to examine multiple cognitive domains, such as visual and verbal memory, executive functions, attention, and spatial navigation. People with cognitive impairment require more time and make a greater number of errors than healthy individuals [65]. For example, patients with cognitive impairment will not be able to remember a list of instructions. The authors mentioned possible limitations if all the adults had similar experiences and functioning in daily life, especially in tasks related to purchases. They also note that the payment in euro currency could have added additional complexity and cognitive load.

The use of VR technologies allows for reduced costs and decreased administration time due to automatic scoring. In addition, participants may be able to self-test in the comfort of their homes without the supervision by a specialist, who will be consulted only if the test detects signs of possible deterioration. However, further studies that include participants with different degrees of familiarity with new technologies, especially tablets, are needed.

#### Computerized Cognitive Games

These applications can be categorized into 2D games and 3D scenario generation. Games (or game batteries) are clinically useful resources that allow for the detection of deficiencies in multiple domains. For example, Episode Gamification [60] is a game that involves taking a virtual walk through a medium-sized city where everyday objects are displayed. The challenge is to remember the maximum number of items to be displayed while avoiding any interfering objects. Another example is Smart Ageing [76], a 3D game that features a loft with a kitchen, bedroom, and living room area. Participants use a touch screen monitor to navigate and interact with the environment, performing five tasks related to daily life: (1) find a list of objects in the kitchen after exploring it; (2) water the flowers while listening to the radio, pressing the space bar each time the word “sun” is heard; (3) make a phone call using the phone book and the phone that are placed on the nightstand, remembering to turn on the television after dialing the number; (4) identify the 12 objects presented in task 1 from a 2D screen with 24 images of objects; and (5) find each of the objects searched for in task 1 while being in the kitchen. Therefore, cognitive computer games have emerged as a novel approach for assessing the cognitive state of people, allowing them to simulate recurring tasks and sensory stimuli while collecting information on the patient’s reaction time in certain tests [60,76].

One problem with several traditional cognitive tests is that they exhibit a “learning bias” [64], meaning that an individual’s cognitive performance improves with repeated exposure to the test, solely because of learning the task, without any actual change in their cognitive ability. Consequently, this bias reduces the reliability of a test when it is used repeatedly (for example, when monitoring performance over time). Computerized cognitive tests can overcome this difficulty by randomly tailoring different task contents to participating adults. In addition, to adopt a psychometric evaluation, it is necessary to normalize the target population according to their specific context and ensure semantic agreement in the tasks, considering both language and culture. For example, in this review, we found only 2 platforms that could be adapted to Latin American population [70,75].

Digital cognitive tests have the potential to be objective, standardized, and most importantly, repeatable. Computerized testing applications provide ideal formats for generating alternative tests, thereby improving test-retest reliability during repeated administration in long-term monitoring. As screening and monitoring tools for serious diseases, computerized cognitive tests are being developed, with emphasis on ensuring their comprehensiveness, validity, and reliability.

Regarding new technologies, although the selected articles were published between 2016 and 2021, only a few used games, virtual or augmented reality, or simulators of daily life for cognitive purposes. Only 4 applications moved in this direction [60,61,65,76], although we expect more to come in the near future. This is especially relevant for cases in which one wants to measure slight cognitive differences over time (whether improvements or deterioration) instead of simply discriminating between a healthy adult, someone with MCI, and a certain degree of cognitive impairment. Our review also found a study that used machine learning techniques [60]. The review by Marques-Costa [55] also observed the need to include item-response theory techniques associated with automatic assessment. The item-response theory can help adapt the difficulty level of cognitive tests to older adults’ personal characteristics and context. This makes us expect a greater inclusion of data analysis and AI techniques in future research, especially those aimed at improving the accuracy of diagnosis and instrument reliability.

With regard to VR technology use, we highlight its absence in cognitive tests. From previous reviews [53-56], the only case mentioned was the Computer Assessment of Mild Cognitive Impairment application, which included a VR driving task [53]. In our systematic review, we found only one study based on supermarkets [65]. It can be argued that this approach allows designers to contrast their batteries with classic paper-and-pencil tests and compare their concurrent validity. At the same time, the higher cost and potential risk associated with developing a VR- or gaming-based test from scratch might also influence the decision to adopt a more “conservative” approach and rely on proven testing instruments.

Finally, an important limitation of most digitized cognitive applications is that they require reliable internet connection. It is necessary for these applications to be able to work offline, saving progress and not depending only on synchronous communication. Future work should explore solutions that can function without a stable internet connection to enhance the accessibility of such tools and encourage their use in rural areas. In addition, in the face of any network contingency, applications that can work offline [72] would perform all its functionalities on a local computer in the face of any network contingency.

### RQ2: What Are the Different Types and Characteristics of Computerized (Digital) Cognitive Tests?

#### Overview

On the basis of the collected information, a characterization of the different digital cognitive tests was conducted to answer the second research question (RQ2). Next, the technical aspects of the tests, such as the administration time, data capture mode, modality, mode of operation (or software instructions), administration method, and location, are detailed. Some criteria or parameters allowed for comparing the different tests included in the selected articles, based on which Table 5 was prepared.

**Table 5:** Main characteristics of the cognitive digital tests.

Cognitive digital test	Time (min)	Input mode	Modality	Software instructions	Exam administration	Location
TOL^a^ [57]	8	Touch Screen, peripheral, or PC mouse	Computer with touch monitor	Visual instructions (text and time limit)	Administered by examiner psychologist	Face to face; laboratory room
eSAGE^b^ [58]	17	Touch screen	Tablet or web-based	Visual instructions	Self-administration	Face to face; community-clinical settings
CoCoSc^c^ [59]	15	Touch screen or headphones	Computer with touch monitor	Visually or verbally (audio)	Self-administered or browser	Face to face; housing and community centers
Episodix Gamification (CVLT^d^) [60]	30-40	Touch screen or PC peripherals (joystick, mouse, and Kinect)	Android or computer (Windows, Linux, or iOS)	Instructions are provided in audio and text format.	Personal clinical support assistance	Face to face; community and university center
SIMBAC^e^ (IADL^f^) [61]	10	Touch screen or PC peripheral	Tablet or computer	Modules with visual (text) and verbal (voice recorder) instructions	Self-managed or trained technician	Face to face; medical care center
UCSF^g^ Brain Health [62]	10	Touch screen	Software platform or iPad 9.7 inch	Instructions in the software; examiner evaluating	Automated scoring; cannot be self-administered	Face to face; diagnosed in university centers
BrainCheack Inc [63]	21 (mean)	Mobile touch screen	iPad, iPhone, or desktop browser	Instruction by examiner	Research staff	Face to face; community center
CGN_ICA^h^ [64]	5	Touch screen	iPad, Raspberry, or web	—^i^	Self-administered or examiner	Face to face or distance; clinic or home
VSM^j^ [65]	25	Touch screen and computer peripherals	Tablet (10-inch) or PC	Instructions by examiner	Self-administered or personal assistance in repeating instructions	Face to face; institutes and medical centers
CompBased-CAT^k^ [66]	10-15	Touch screen, PC peripherals, or headphones	Tablet (Asus) or computer (Windows 10)	Visual on-screen instructions and voice with external noise-cancelling hearing aid	Self-administered or minimal assistance in instructing	Face to face; institutes and geriatric hospitals
CogEvo [67]	10	Touch screen	Computer OS^l^	Audiovisual with home icon	Administered by examiner	Face to face
CANTA^m^ [68]	45-60	Touch screen	Touchscreen computer (Windows)	Verbal instructions from the instructor	Administrated with trained supervisor	Face to face; adult centers
Cognivue [69]	10	PC peripherals	Computer OS	Automated instructions and test subbattery	Self-administered or assisted by nonclinical support staff	Face to face or clinical establishment. No specific place
BHA-CS^n^ [70]	10	Touch screen	Software and tablet, TabCAT Pad de 9,7	—	Managed by examiner	Face to face; adult and Alzheimer centers
Inbrain CST^o^ [71]	30	Touch screen	Tablet or OS Microsoft Windows 10	Verbal (written)	Minimum attendance	Face to face or distance; private room in clinic
Trail Making Test y Bells Test [72]	5	Touch screen	Tab A6 con S pen or Webserver nube	Verbal assistant, virtual supervision of the test	Unsupervised environment; virtual only	Distance; Geriatric Foundation or Home
C-ABC^p^ [73]	5	Touch screen	Computer (OS) with touch screen (80×60)	On-screen text and verbal description with headphones on PC	Assistance by a psychologist if needed	Face to face; Memory Clinic
EC-Screen^q^ [74]	5	Touch screen	Web or tablet	Reading questions answer	Autoadministrated or assisted	Face to face or distance; geriatric community settings
BHA [75]	10-5	Touch screen	Tablet or web	Digital survey	Neurologist examiner or neuro psychologist	Face to face or institute community centers
SG^r^ (IADL) [76]	10-30	Touch screen	Touch screen computer	Visual instructions (and examiner)	Administered in the presence of a neuropsychologist	Face to face or neuropsychology unit, communities

^a^TOL: Tower of London.

^b^eSAGE: Self-Administered Gerocognitive Examination.

^c^CoCos: Computerized Cognitive Screen.

^d^CVLT: California Verbal Learning Test.

^e^SIMBAC: Simulation-Based Assessment of Cognition.

^f^IADL: Independent Activities of Daily Living.

^g^UCSF: University of California, San Francisco.

^h^CGN-ICA: Cognitivity Neurosciences-Integrated Cognitive Assessment.

^i^Not available.

^j^VSM: Virtual Supermaket.

^k^CompBased-CAT: Computer-Based Cognitive Assessment Tool.

^l^OS: Multiple Operating System

^m^CANTAB: Cambridge Neuropsychological Test Automated Battery.

^n^BHA-CS: Brain Health Assessment-Cognitive Score.

^o^CST: Cognitive Screening Test.

^p^C-ABC: computerized assessment battery for cognition.

^q^EC-Screen: Electronic Cognitive Screen.

^r^SG: serious game.

#### Time

The administration time of the tools found in the literature varied from 5 to 45 minutes. Compared with the traditional MMSE assessment that relies on professional training, digital cognitive tests take approximately 10 minutes, excluding the time needed to score the participant. In contrast, if the examination is performed by a technician or someone without specialized training, it will take much longer [66]. Computerized batteries allow for more accurate measurements; for example, the computerized assessment battery for cognition [73] can be administered in a short amount of time (approximately 5 min). It is a sensitive battery that detects not only cognitive impairment but also dementia. Finally, positive results should be considered supportive methods and not definitive diagnoses. Therefore, patients should be referred for a more comprehensive evaluation by health professionals [59].

#### Input Mode (or Capture)

The data-acquisition mode was identified using a touch interface and computer peripherals. In a study of digital games, it was found that older adults preferred a touch interface to computer peripherals such as a keyboard and mouse. In addition, in the digital version of the Self-Administered Gerocognitive Examination (eSAGE) [58], the participants did not use a stylus but instead used their fingers to draw or write the requested answers. Finally, the use of hearing aids allows for the cancellation of external noise and helps patients avoid distractions [66].

#### Modality (of the Device)

Psychometric instruments were implemented on desktop computers, laptops, tablets, iPads or iPhones. The use of larger screens helps individuals with visual impairments access and complete the tests. These digital methods allow us to capture the response time with better precision. For example, the Electronic Cognitive Screen [74] integrates the clock task, which reflects the speed of processing, and executive function. This test, when displayed on a tablet, allows for the detection of a person’s lesser fine motor control; it is easier than the paper version for older adults.

#### Software Instructions

The mode of operation (or software instructions) can be entered by examiners or more easily integrated into the software. For the “Simulation-Based Assessment of Cognition” simulation software, the instructions consist of voice recordings and text files [58]. Each module incorporates specific instructions. The platforms read the questions and then prompt the participants to select the correct answer. The “TMT and Bells” tests [72] incorporate AI to detect the participants’ voices and dictate the task guidelines. In the virtual game “VSM,” the guidelines are shown visually and auditorily on the screen for each activity in the game’s virtual environment [65]. The administration of the digital version is minimally assisted because each item comes with simple written instructions. Finally, the role of the examiner must be clear because providing clues during the test can introduce bias into the evaluation.

#### Test Administration

The administration of a psychometric instrument can influence the results. The advantage of administering digital cognitive tests in a laboratory is the potential to reduce the frequency of errors, software failures, and interruptions, but the psychometric instrument can be conditioned to exclude patients [57]. The Computerized Cognitive Screen [59] allows for self-administration with automated scoring while still requiring minimal assistance for older people. The automated calculation of scores in the “Cognivue” battery [69] is more efficient and consistent than traditional tests. In Episodix [60], standardized administration makes data collection and response time capture more efficient. In the digital version [58], web-based administration is useful for people living in rural regions with limited resources and a lack of access to health care providers. The test supervisor only observed and served as a guide or security during the evaluation. The greatest advantage found in VR environments is allowing older adults to check their cognitive functioning at home and only visit a specialist if necessary [65]. The Integrated Cognitive Assessment visual categorization test [64] was found to be self-administered due to its simple design with no language and culture barriers. The platform [66] has an automatic scoring algorithm that helps alleviate the burden on the professional staff. Thanks to automatic scoring, not requiring individuals with specialized knowledge makes it easier to eliminate human error and reduce the duration of examinations.

#### Location (or Place)

Participants were recruited from clinics, memory study centers, hospitals, and neurological institutions. Diagnostic tests in community centers are beneficial because they allow digital cognitive tests to be performed on a larger scale and are easily accessible, as they require minimal professional demand. In addition, for some older adults, interacting with a computer can be more pleasant than visiting a health care facility. It can also be administered by individuals with a lower level of education, although guidance from professionals is necessary for those in community centers and primary health care clinics. Home-administered cognitive tests can provide a relaxing, nonintrusive, and familiar environment for patients [64,72]. The flexibility of the applications in their electronic versions also facilitates their administration in the comfort of one’s home using a mobile device or tablet. In the case of the TMT-Bell digital test [72], the authors emphasized the need to strengthen the collaboration between technicians and clinicians. They also suggested the development of novel indicators that could further enhance the utility of digitized tests. In addition, digitized tests were performed in a clinical setting under the supervision of a trained professional. Therefore, they proposed conducting more usability and validity studies on their entire platform in a domestic environment. Despite the digitization of cognitive tests, only 4 applications have declared that they can be used remotely (web-based) without the presence of a supervisor [64,71,72,74].

From the reviewed articles, we can conclude that there are clear advantages to conducting (digital) tests using technological devices. These tests allow psychometric evaluations to be executed in the comfort of one’s home or community centers [62,63,65,70,73-75], with minimal intervention from a specialist professional. In addition, most psychometric tests were implemented on equipment with touchscreen devices, such as notebooks, tablets, and smartphones. However, there was one exception: the Cognivue application [69] was carried out with a desktop computer. In cases where an older person is not familiar with technology, assistance can be provided by a family member or the clinical supervisor.

In addition, computerized cognitive tests provide new opportunities to remotely monitor cognitive changes and detect early dementia. Without the need to visit a trained practitioner or counselor, computerized cognitive tests can be distributed over the internet and self-administered [58,59,66,71,72,74] or eventually supported by a supervisor [61,64,65,69]. Some tests need to be administered by neurologists, psychologists, or specialized clinical personnel [57,60,62,75,76].

Technology benefits psychometric tests in numerous ways. For example, it significantly reduces the time that elapses between the application of the evaluation instrument and the reporting of results, sometimes providing immediate feedback. In addition, technology helps to eliminate qualification errors that are common among human beings. It is also possible to implement technological security measures, such as using passwords, and finally, it enables the adaptation of the content of the test according to the characteristics of the person who will answer the test [32].

In addition, in the field of psychology, there is a growing need for cultural adaptation tests to prevent or minimize errors that may introduce bias, such as construct, methodological, and item-related errors, resulting from improper use of the tests. Therefore, it is necessary to thoroughly consider language and cultural differences. This will ensure that valid and reliable measurements can be obtained when the tests are applied to different cultural groups [77]. For example, in one study [75], the Brain Health Assessment (BHA) was translated and adapted into Cuban Spanish by a multidisciplinary team of four language experts: 1 geriatrician, 1 neurologist, 1 psychiatrist, and 3 neuropsychologists. The adaptation was performed in accordance with the current guidelines for cross-cultural test development, with the Guidelines for Translating and Adapting Tests [78,79], and the results were validated with the MoCA. In particular, verbal stimuli on the favorites task were adapted to have similar frequency and complexity as the English version and to represent culturally appropriate concepts in the Cuban context. Furthermore, the video game Episodix [60], which has a multilingual design and is supported in English, Spanish, and Galician, was adapted and administered in Spain; the Spanish version of the California Verbal Learning Test (CVLT) is known as test de Aprendizaje Verbal España-Complutense in Spanish [80], and their results were validated with the MMSE.

### RQ3: What Are the Main Characteristics of the Subtests and Tasks Used in Different Digitized Cognitive Tests?

A test could be intended to assess a specific cognitive function or several functions, and a particular cognitive function could be assessed using one or more tasks. Table 6 shows the number of articles in terms of the evaluation of cognitive tasks in each application or test. Of the 20 articles, digital applications that included up to 5 different tests were found [61,67,68,76]. Six tests were found in the studies by Wong et al [59] and Takahashi et al [66], whereas 7 tests were found in the studies by Scharre et al [58], Groppell et al [63], and Chin et al [71]. One article included 8 different tests [73], and another article included up to 10 tests [69]. When examining articles that included a task or activity to assess different cognitive functions (Table 7), it is evident that executive functions and memory are the most commonly addressed, whereas praxis, abstractions, and calculations are less frequently discussed. Regarding memory and its types (Table 8), we found that memory in general was the most extensively covered, assessed by 6 articles, whereas remote and prospective memory were the least covered, assessed by 1 article each.

To answer the third research question (RQ3), we examined the operational performance of all the digitized cognitive tests. Table 9 lists the applications discussed in the 20 articles, with a more detailed overview of the work carried out. This table includes the digital applications and their respective cognitive domains that they evaluate and train. It also provides a summary of how each application works and the sequence of tasks required of the participant.

**Table 6:** Papers by the number of task of the digital test.

Number of task of the digital test	Papers (n=20), n (%)
1	4 (20)
2	0 (0)
3	2 (10)
4	3 (15)
5	4 (20)
6	2 (10)
7	3 (15)
8	1 (5)
9	0 (0)
10	1 (5)

**Table 7:** Papers by assessed cognitive functions.

Functions	Papers (n=20), n (%)	References
Executive functions	16 (80)	[57-59,61-63,65-68,70-72,74-76]
Memory	15 (75)	[58-60,62-68,70,71,73-75]
Visuo-spatial	10 (50)	[58,62,65,67,69-71,74-76]
Attention	8 (40)	[59,65-68,71-73]
Language	6 (30)	[62,63,70,71,75,76]
Orientation	6 (30)	[58,59,67,69,71,73]
Processing speed	6 (30)	[62,64,68-70,72]
Learning	2 (10)	[59,64]
Praxis or gnosis	1 (5)	[63]
Abstractions	1 (5)	[58]
Calculations	1 (5)	[58]

**Table 8:** Papers by types of evaluated memory.

Memory types	Papers (n=20), n (%)	References
Memory (general)	6 (30)	[59,62,63,67,71,74]
Work memory	3 (15)	[59,68,75]
Episodic memory	3 (15)	[60,70,75]
Visual memory	2 (10)	[64,65]
Intermediate memory	2 (10)	[66,73]
Verbal memory	2 (10)	[58,65]
Remote memory	1 (5)	[66]
Prospective memory	1 (5)	[59]

**Table 9:** Type, domains, and description of tasks of digital applications.

Name of the evaluation, test, or battery; cognitive domains; type	Cognitive assessment tests, n	Brief description by activity
Computerized TOL^a^ [57]; planning as an executive function; specific	1	Tower task (rule breaks and errors during planning): the TOL consists of a series of instructions using colored balls (red, yellow, and blue) that are suspended within 3 bars. A reference configuration is required to be completed with a minimum of movement. Only one ball can be moved at a time; in each bar, only the most prominent ball can be moved, and the balls are only deposited on the bars. The examiner explains the instructions and before starting the test, presents a set of experimental problems to familiarize the participants with the task; in addition, there is a time limit of 1 min per test, with 24 tests. The software records the planning error when the participant tries to select a blocked ball that is under another ball, places a ball on a bar that has the number of full balls, or selects a position other than the final goal.
Digital Gerocognitive Examination (eSAGE^b^) [58]; orientation, executive-level, memory language, abstraction, calculations, and visuospatial skills; screening	7	Date: the participant is instructed to write the date of the day. Picture naming and verbal fluency: words are presented backward, and these describe the names of the images that appear on the screen (eg, piano and volcano). Delayed recall and written instruction: the instructions for the tests must be memorized, and at the end of the last page, “I finished” must be written. Modified Trail B and problem-solving task: they are then asked to draw a line from one circle to another, starting at 1 and alternating numbers and letters in order before ending in the letter (1, A, 2, B, 3, C, etc). Determining similarities: later, they are asked to answer questions by comparing objects. Word problem calculation: a problem must be solved with letters. Copy 3D construction and clock drawing: a 3D square should be copied, and a clock face is drawn with the digits from 1 to 12. Then, the hands for 5 min past 11 o’clock (11:05) are asked to be drawn, and finally, the individual should place an L on the hour hand and an S on the min hand.
CoCoSc^c^ [59]; learning and memory, executive functions, orientation, attention and working memory and PM^d^; screening	6	Time-based PM and even-based PM: participants are instructed to perform an objective action; then a digital clock is displayed on the screen to control the time. The clock disappears if there is success, or after 30 s if no action has been executed. In the second PM test, the same procedure is followed but with different objectives. Conflict inhibition (ringing doorbell): patients are asked to touch the screen twice in response to the sound of a doorbell and only once to the double sound of the doorbell (6 practice tests are administered before the evaluated activity). Word List Learning: a list of 6 words of 2 syllables is presented at a speed of 1 s, and then the person remembers the word and mentions it aloud, thus facilitating the encoding of the memory. Orientation to year, month, and day of the week and orientation to place: chronological orientation is measured by sets of questions and selection answers (“Yes,” “No,” “I don’t know”), then answers to questions from the region, district, residential area, and location of the test are chosen. (“I don’t know” and “Don’t” are incorrect answers.) Attention and working memory (test analogous to the Wechsler memory scale): the participant must reproduce on the screen of the device the sequential order (forward and backward) of the location where rabbits appear. Delayed word list memory: there are 12 words of 2 syllables, which are classified into objective and distracting words; the participant indicates whether the word is objective or distracting.
Episodix, CVLT^e^ [60]; episodic memory, regarding the verbal; specific	1	CVLT: it is a game based on learning and a memory word list from the CVLT test. It covers many more elements of episodic memory than just those involve remembering words verbally. The CVLT is a word set task that is displayed as a shopping list for a day of the week, and the participant performs the test and must remember all the items on the list. A second list of interferences is then presented for the next shopping day. After waiting for a while (the recovery phase) and producing a third list that includes 2 d of the week with new items, the participant must recognize the items in the list. The game is presented in a virtual village, where the participant moves to find objects that are displayed visually and audibly. There are 3 collections of objects; as in the original test, they are denoted as lists A, B and C, where list A allows the main learning, list B is interference, and list C is the recognition list. In addition, the recovery phase is time based, similar to the CVLT.
SIMBAC^f^ [61]; IADL^g^ executive functions; specific	5	Facial recognition and memory of names: an image of a human face is observed for 5 s, followed by a series of facial images of different sexes and ages. The image is paired with a unique name. Then several images are displayed, and the individual must select the photo that is paired with the image seen and the name. Six trials (3 males and 3 females) are presented; the difficulty grows with 2 to 4 name and face trials. Filling a pillbox: a 7-d pillbox with 14 compartments is shown; in 3 pillbox containers, the instructions “take a pill overnight” are explained. They must correlate the shape and color of the pills within the pillbox, selecting the pill with the corresponding medication compartment. Using an ATM^h^: the instructions indicate that money is withdrawn from a virtual ATM. An ATM card must be inserted, the PIN and the amount to be withdrawn must be entered, the money is withdrawn, and the card is recovered. Automated prescription renewal by phone: participants are requested to renew the prescription of a drug in a pharmacy over phone using the drug label and the call system. The participant taps the phone and listens to a voice recording with instructions.
UCSF^i^ BHA^j^ [62]; memory, executive function, processing speed, visual-spatial, and language; screening	4	Favorites (memory domain): participants are asked to indicate their favorite people, foods, and animals, and then they are shown 4 different faces 2 times. Each face is paired with a favorite food or animal. The pair is shown to them for 5 s and will reappear in random order. After each learning test, faces reappear, and participants are asked to remember the food and animal associated with each face. Match (executive and speed domain): participants were shown a fixed caption of numbers 1 through 7, with corresponding simple abstract images appearing just below each number. The participant was instructed that whenever a number appeared in the middle of the screen, he should tap the corresponding image at the bottom of the screen as quickly as possible. After each answer, a new number appeared. Accurate responses were totaled in 2 min. Line orientation (visuospatial domain): participants were shown 3 lines on a black background, a 2×50-mm white line shifted vertically above two 3×25-mm orange lines. One orange line was parallel to the white line, and another line was at a different angle. The white line was randomly placed at any angle between −60° and +60° from the vertical. Participants were asked to hit the orange line, which was parallel to the white line. The difficulty was manipulated from test to test by varying the angle difference. Animal fluency (language; widely used test): participants name different animals as fast as they can for 1 min.
BrainCheck memory assessment Inc [63]; memory, language, praxis, gnosis, and executive functions; screening	7	They are standardized tests of psychometric manuals. Immediate recall: A stimulus is presented to the participant, and he is asked to remember it immediately after having presented it. Delayed recall: the participant will be given some material to remember, either a list of words or a paragraph. Moreover, at a later time, individuals will be asked to remember those items. TMT-A^k^: participants are asked to draw a line connecting 25 numbers in sequential order. TMT-B^l^: this requires participants to connect, in sequential and alternating order, 13 numbers and 12 letters (1, A, 2, B... N and 13). The indicator is the error when connecting the sequence and the time to perform the action. Stroop task: the names of the colors were displayed incongruously; for example, the color red was written in blue font. Participants had to say the color of the font and not read the word, among several options. The total number of correct answers for each of the 20 items in the test was recorded. Digital symbol substitution task: consists of pairs of digits and symbols (eg, 1/-, 2/┴ ... 7/Λ, 8/X, 9/=) followed by a list of digits. Below each digit, the participant must write the corresponding symbol within the allowed time. Matrix problems task: boxes are shown with certain schemes or figures, in which the individual has to add a missing part, and the individual must choose that part from several options.
CGN_ICA^m^ [64]; it correlates with processing speed, learning and visual memory; screening	1	ICA test: it is a visual characterization task that presents on-screen 100 black-and-white images of animals and places. Each image is displayed for 100 ms, followed by an interval of 20 ms, then a mask with white noise for 250 ms, and to finish, the participant must respond to the image he or she observed. The speed and accuracy of the test are quantified. Four experiments were performed, and only 2 were considered for our study.
VSM^n^ [65]; visual memory and verbal, executive functions, attention, and spatial navigation; screening	1	VSM is based on a daily buying activity. A shopping list appears in the upper-right corner of the screen during exercise. The person is expected to locate the items on this list, place them in the shopping cart, take them to the checkout counter, and pay the correct amount for purchases. The participant is asked to move the shopping cart and navigate inside the VSM by tapping green footprints on the screen. It is an exercise designed to examine multiple cognitive domains (as noted).
CompBased-CAT^o^ [66]; attention and concentration, immediate memory, remote memory, executive function, and selective attention and perception of space; screening	6	Digit span forward and digital span backward: In task 1, participants were asked to immediately remember a set of numbers that were presented in random order on the PC screen. In task 2, participants remembered the same set of numbers in reverse order. The number of digits gradually increased (3, 5, 7, 8). The maximum number of digits a participant could remember in the correct order (forward or backward) was recorded. Memory of item names and tasks and memory recall of item names: In task 3, participants were asked to memorize 10 target words that were presented on the PC screen. Subsequently, 20 words were shown, including 10 target words and 10 distracting words, and participants were asked to select the 10 target words. This was repeated at 2 attempts, and the number of correct answers was recorded on the second attempt. In addition, participants were instructed to remember the 10 target words after all other tests, and the total number of remembered target words was recorded. Stroop task: the names of the colors were displayed incongruously; for example, the color red was written in blue font on the PC screen. Participants had to say the color of the font and not read the word among 4 options. The total number of correct answers for each of the 20 items of the task was recorded. Figure recognition task: in task 6, the blocks were esthetically stacked and displayed on the PC screen. Participants were asked to indicate the number of blocks. We recorded the total number of correct answers for each of the 8 items in the task.
Cognitive Function Balancer (CogEvo) [67]; orientation, attention, memory, executive function, and spatial cognition; specific	5	Orientation: it is the task of selecting the day, week, and time of the exam and randomly displaying options for questions of the day, week, and time. Follow the order: the purpose of the task is to select numbers or letters, then touch the screen again, but this time alternating digits with characters according to the logical order. For example, 1, 2, 3; ABC and alternates 1, A, 2, B, 3, C. In total, each question consists of 6 digits, 12 characters and 8 combinations. Flashing light: a random pattern of lights (red, blue, green, and yellow) must be memorized to follow a certain sequence. The test difficulty level increases, up to 16 combinations of lights that depend on the correct answers. The score is calculated based on the response rate and accuracy. Route 99: squares are drawn from the starting point to the goal, followed by digits shown at random from 1 to 10. It is forbidden to pass obliquely or travel through the same area. It can be 16, 36, or 64 squares (N×N). Same shape: the task is to choose a central figure of 6, which is located around. A total of 4 questions are selected, and for each question, a circle must be marked in the figure.
CANTAB^p^ [68]; attention and working memory, psychomotor or processing speed, and executive functions; specific	5	The rapid visual processing subtest: a sustained attention test involving a series of numbers that progress steadily over 6 min, during which the participant must press a button each time a specified 3-digit sequence occurs. The spatial working memory task: tests patients’ ability to retain spatial information and manipulate items remembered in working memory and to do so strategically. It requires the participant to “open” a series of colored boxes to find a tab and then perform a new search to find the next tab. The reaction time index: measures the amount of time that elapses between the appearance of a stimulus on the screen and the release of a button by the participant (reaction time) and from releasing the button to touching the screen (movement time). The delayed matching task: it involves a visual pattern that is displayed and then removed, and after an interval of 4-12 s, 4 patterns are displayed, from which the participant must choose the pattern that matches the initial pattern. The paired frames learning task: it is a series of boxes that appear on the screen, each of which “opens” in turn, and some of which contain a pattern. Subsequently, the participant is shown the patterns and must select the boxes that contain them.
Cognitive Assessment Test (Cognivue) [69]; scores correlate with domains of verbal processing, speed, visual acuity, visuospatial function, orientation, and sequencing; screening	3	Adaptive motor control test: evaluates visuomotor responsiveness using speed and accuracy measurements (adaptive motor control test and visual salience test). Perception processing: measures the perceptual processing of the participant in different ways despite adding increasing patterns of disorder. Letter discrimination: the participant must discriminate real English letters from others that are similar in a variety of ways to those letters. Word discrimination: the participant should discriminate real 3-letter words from 3-letter nonwords. Shape discrimination: discriminates a circle filled with a common shape from the rest of the screen filled with other common shapes. Motion discrimination: discriminates a circle filled with one direction of point movement from the rest of the full screen with another direction of point movement. Memory processing: evaluates memory using specialized sets of visual stimuli. Letter memorization: measures the participant’s ability to remember which letter was presented as a preindication and then select that letter from a sample of alternative elements, despite the addition of increasing amounts of clutter, and the participant must select the correct letter of the English alphabet. Word memory: select the correct 3-letter word. Shape memory: select the correct shape. Motion memory: select the correct direction of movement.
BHA-CS^q^ [70]; episodic memory, executive functions, processing speed, language generation and visuospatial; screening	4	Favorites (memory domain): measures immediate verbal and visual associative memory and delayed visual memory. Participants are asked to remember people and their favorite foods and animals. In each of the 2 learning tests, 4 different faces were shown twice, once with a favorite food and once with a favorite animal. Accuracy is assessed through 2 immediate recovery attempts and a 10-min delayed recovery test. Match (executive and speed domain): a series of digits is displayed in the center of the screen, and participants are asked to tap the corresponding image at the bottom of the screen as quickly as possible. Accurate responses are totaled in 2 min. Line orientation (visuospatial domain): several lines are displayed on the screen, and participants are asked to tap the orange line, which is parallel to the white line. The “angle difference” between the mismatched orange line and the white line is scaled based on the accuracy of the answer, and the scores estimate the angle difference when the probability of a correct answer is between 71% and 75%. Animal fluency (language): measure categorical verbal fluency. Participants are asked to name as many animals as they can in 1 min.
Inbrain CST^r^ [71]; attention, languages, memory (and temporal orientation), executive function, and visuospatial; screening	7	VST^s^: Nine squares are located on the screen. The squares briefly change color in sequence from 2 to 8, and participants must touch the figure in the same order (task forward) or reverse order (task backward). One point per correct sequence is added, and the score range varies from 1 to 14. The task stops when the participant fails twice. DNT^t^ (confrontation names): it is a task of 15 drawn elements that are relatively difficult to pronounce due to their low frequency of use. Semantic test: the participant lists as many fruits as possible, and Phonemic test: the participant must list as many words as possible, in both cases, for 1 min. Block design: a pattern is displayed on the screen, using 6 types of 2-color squares or a combination of them. The participant must reproduce the same pattern by dragging one of the 6 squares. There are 10 patterns, and the difficulty depends on the number of squares and time. Spatial orientation: the participant must indicate the year, month, date, and day of the week of the current period. WPAT^u^: Nine words are displayed in a 3×3 grid in a given sequence. The examinee must memorize the words and their location in the grid, and then the participant is asked to remember the words immediately and after 10 min and to recognize both the word and its location. K-TMT- A: the participant is asked to connect numbers (1-15) in ascending order as quickly as possible, using an “S pen” on the tablet. The time taken to complete the test is measured. K-TMT-B: the participant is asked to connect numbers and the day of the week, alternately and in order, as quickly as possible.
Trail Making Test y (Bells Test) [72]; psychomotor speed and mental flexibility and executive functions and attention; screening	3	TMT-A: participants are asked to draw a line connecting 25 numbers in sequential order. TMT-B: requires participants to connect, in sequential and alternating order, 13 numbers and 12 letters (1, A, 2, B... N, and 13). The indicator is the error when connecting the sequence and the time to perform the action. Bells: this test allows the assessment of the attention span through a visual search task; the participant is asked to search and mark 35 objects (black ink bells). The indicator is the number of correct targets.
New C-ABC^v^ [73]; sensor-motor ability, attention, orientation, immediate memory, and an arithmetic problem; screening	8	Touching a moving target: the circle lens was presented in different places on the screen, one at a time, and the participant was asked to touch the circle lens as quickly as possible. The digits order: 9 digits (1-9) were presented in random positions on the screen, and the participant was asked to tap the digits in sequential order as quickly as possible. Time orientation: the participant was asked to choose today’s date (day, month, year, Japanese era name, and day of the week) from a list of candidates on the screen. The letters-recognition memory: 4 Japanese letters of hiragana (“ri,” “na,” “ku,” and “me”) with meaningless relationships were presented on the screen for 5 s. The participant was then asked to select the 4 recognized Japanese letters from the syllabary. The numbers-recognition memory: 3 numbers without serial numbers were presented one by one on the screen. After 5 s, the participant was asked to select the 3 numbers in the correct order on the license plate. The figures-recognition memory: 4 figures with different conditions in color and shape are presented on screen. After 5 s, the participant was asked to select the 4 recognized figures from a set of 12 candidates. The arithmetic problem: a shopping story was presented on the screen; the participant was asked about the total number of products purchased at 2 stores, and then the participant was asked to select the correct number from a set of options. Detecting a digit test: a table of random sequences of digits is presented on the screen. It is asked to detect and touch the digit (3) of the table, and there are 4 elements of digit 3.
EC-Screen^w^ [74]; executive functions, visuospatial skills, mental flexibility, and memory function; screening	3	Clock test: requires the participant to place a specific time by moving the hands of a digital clock. Proof of history: it is a fact-conversion test based on the history of a known landmark in the country. The platform reads a short story, and the participant must remember the facts and identify the facts based on questions. Deferred recognition test: the participant must learn and remember 5 words from 2 syllables that are read by the software.
BHA [75]; episodic memory, attention and working memory, executive functions, visuospatial skills, and language; screening	4	The same tests on BHA battery were already mentioned [70]: Favorites (memory domain): measures immediate verbal and visual associative memory and delayed visual memory. Participants are asked to remember people (faces) and their favorite foods and animals. Accuracy is assessed through 2 immediate recovery attempts and a 10-min delayed recovery test. Match (executive and speed domain): a series of digits is displayed in the center of the screen, and participants are asked to tap the corresponding image at the bottom of the screen as quickly as possible. Accurate responses are totaled in 2 min. Line orientation (visuospatial domain): several lines are displayed on the screen, and participants are asked to tap the orange line, which is parallel to the white line. The scores estimate the “angle difference” between the mismatched orange line and the white line when the probability of a correct answer is between 71% and 75%. Animal fluency (language): measure categorical verbal fluency. Participants are asked to name as many animals as they can in 1 min.
Smart Aging Platform (serious game; IADL) [76]; executive, verbal, spatial function; screening	5	It is a game where the participant experiences a virtual environment resembling a loft; the objective of the activity is to perform tasks, and the system records the data of the positions, times, actions, etc. To quantify cognitive functions, the system provides scores and calculates indexes (accuracy, time, and distance). Time is timed from start to finish. The distance is the number of meters traveled. Usually, people who take longer to perform tasks have a diagnosis of mild cognitive impairment. Object search: the participant is asked to search for a list of objects. Water flowers while listening to the radio: participants are asked to turn on the radio and press the space bar each time they hear the word “sun” while watering the flowers. Make a phone call: the participant is asked to make a phone call using the phone book and then turn on the television. Choose the right object: a screen displays 24 images of objects. The participant must identify the 12 objects presented in task 1. Find the objects: the participant is placed in front of the kitchen and asked to look for each of the objects.

^a^TOL: Tower of London.

^b^eSAGE: Self-Administered Gerocognitive Examination.

^c^CoCosc: Computerized Cognitive Screen.

^d^PM: prospective memory.

^e^CVLT: California Verbal Learning Test.

^f^SIMBAC: Simulation-Based Assessment of Cognition.

^g^IADL: Independent Activities of Daily Living.

^h^ATM: automated teller machine.

^i^UCSF: University of California, San Francisco.

^j^BHA: Brain Health Assessment.

^k^TMT-A: Trail Making Test-A.

^l^TMT-B: Trail Making Test-B.

^m^CGN_ICA: Cognitivity Neurosciences-Integrated Cognitive Assessment.

^n^VSM: Virtual Supermarket.

^o^CompBased-CAT: Computer-Based Cognitive Assessment Tool.

^p^CANTAB: Cambridge Neuropsychological Test Automated Battery.

^q^BHA-CS: Brain Health Assessment-Cognitive Score.

^r^CST: Cognitive Screening Test.

^s^VST: visual span test.

^t^DNT: difficult naming test.

^u^WPAT: word place association test.

^v^C-ABC: computerized assessment battery for cognition.

^w^EC-Screen: Electronic Cognitive Screen.

The different applications also differ depending on whether the tests are of the screening type or are evaluation tests of specific cognitive domains. The number of tests varies depending on the domains required for clinical evaluation. In general, at least 2 cognitive domains, such as language, praxis, gnosis, or executive functions, are necessary to identify dementia. In addition, a test can involve subdomains. For example, the Episodic Assessment is a modified version of the CVLT and incorporates gamification techniques to assess episodic memory. The advantage of the game is that it allows for the coverage of more elements of executive function rather than those involving simply remembering words verbally [60,63,64].

### RQ4: What Are the Main Effects, Personal Traits, and Psychometric Parameters Considered for the Validation of Digitized Cognitive Tests for Older Adults?

#### Main Effects

All the articles focused on the capacity and accuracy of the test to measure the cognitive level of the patient. However, it has been overlooked in most cases that unlike paper-and-pencil tests, computerized cognitive tests are software programs. Therefore, usability (ease of use) and human-computer interaction dimensions are crucial to their overall effectiveness. Only 2 articles from the review used usability questionnaires [60,72]. Thus, the inclusion of usability tests in cognitive evaluation software is expected in future work, either through usability tests with users or through the evaluation of experts through appropriate usability heuristics.

In addition, digital tests could be affected by the level of digital literacy of older adults or by their aversion to new technologies. People have different levels of skills and use of information and communication technologies, which makes it necessary to assess the extent to which they accept technology. Depending on the user, the degree of information and communication technology acceptance can positively or negatively affect the use of a particular piece of software, such as a shopping website, a video game, or, in our specific scenario, a digital cognitive test. Although this factor may be more relevant when dealing with older adults, it is worth noting that only one of the analyzed studies [72] addressed this aspect. It would be interesting to include the Model of Acceptance and Adoption of Technology in Older Adults, known as Senior Technology Acceptance Model [81,82], in future studies. This model measures various aspects such as intention to use, perceived usefulness, ease of learning, and actual use. By evaluating how diverse levels of technology acceptance affect digital cognitive test performance, we can gain valuable insights.

Other related effects that can be measured in older adults include psycho-emotional aspects such as motivation, effort, tension, and anxiety. On the one hand, there is the overall emotional level of the person, and on the other hand, there is the emotional level that is generated when using a cognitive digital test. One factor to consider is an individual’s level of motivation on a given day. Another factor is the degree of motivation specifically for taking a digital cognitive test, as well as the level of effort exerted when performing each task within the test. The study by Valladares-Rodriguez et al [60] included a motivation test and that by Eraslan Boz et al [65] applied a test for anxiety and depression, but only descriptively. They did not evaluate the effects of these emotional factors on cognitive test performance nor did they compare the results between conditions with different levels of anxiety, motivation, or depression. Therefore, we consider it important to measure and evaluate the possible moderating role of diverse emotional factors in future studies, particularly when examining games, simulators, and other immersive software for cognitive evaluation.

Another aspect that has received little attention is the accessibility of software, including sensory (vision and hearing) and physical accessibility. It is necessary to consider potential users who may have visual impairment, illiteracy, or movement disorders that can make it difficult to administer the digital test through a tablet or mobile device [63].

#### Personal Traits

Regarding the main personal traits considered in cognitive tests, the first criterion is to group the participants based on their cognitive ability (eg, normal, MCI, and dementia). Previous surveys mentioned the need to cover the different personal traits of older adults. The most frequently mentioned traits were age, educational level (years), and gender. However, less attention has been given to language, ethnicity, and cultural aspects. For example, the review by Aslam et al [54] includes a comprehensive analysis of the population, considering cognitive level and nationality as well as demographic factors such as age, gender, and educational level. The review by Zygouris and Tsolaki [53] highlights the difficulty that some tests are not adapted for languages other than English and that the results of certain tests are influenced by an individual’s educational level. Tsoy et al [56] includes statistics about the age and years of education of the selected papers but does not consider sex or cultural aspects, although it does highlight that the different tests are not available in several languages. The survey by Marques-Costa et al [55] included data about age, gender, and educational level, but this information was presented in narrative form rather than a comparative table. This suggests that further research is needed to examine specific patient populations of different age groups and educational levels. In addition, the review by Chan et al [83] does not include demographic or educational data.

When analyzing the reviewed papers in our study, we categorized the 20 papers into 5 groups. The first group [60,62,63] did not include any demographic variables. The second group [57,66,72,76] includes demographic and comparative tables of results by cognitive condition but lacks further analysis or discussion of the participants’ demographic characteristics. Other studies (third group) conducted an initial analysis of the personal traits of older adults without making statistical adjustments or comparing these characteristics with associated groups. For example, the study by Khaligh-Razavi et al [64] included a demographic table and raised the issue of test dependency on the level of education. In addition, Cahn-Hidalgo et al [69] mentioned that there were biases related to education, language, gender, and culture, but they claimed that their proposed cognitive test was free from these biases. However, no further empirical evidence was provided to support these test features.

A fourth group of studies statistically adjusted the results of the cognitive tests based on demographic characteristics. For example, the studies by Scharre et al [58], Rapp et al [61], and Eraslan Boz et al [65] included a demographic table and considered adjustments based on age and education. However, the study by Rapp et al [61] did not include the years of study in the table. In the study by Schulz et al [68], the results were adjusted for age using Heaton-Revised Norms, while the study by Chan et al [74] adjusted for participants with fewer years of education and provided an “illiterate” version of the test, allowing for more time during administration. In addition, Rodriguez-Salgado et al [75], on the basis of sample controls, they adjusted for age, education, and sex using a regression-based approach, arguing that assessments are complicated by linguistic, ethnic, cultural, and socioeconomic diversity.

Finally, the fifth group performed statistical comparisons by considering different demographic characteristics (older adults’ traits). For example, Wong et al [59] presented a comparative table showing the results of cognitive subtests based on the level of education. Ichii et al [67] compared test results based on age and cognitive groups. Eraslan Boz et al [65] evaluated the effects of age, gender, and education. Tsoy et al [70] considered adjustments for age, education, sex, and language, in addition to performing a linear regression considering sex and age. In the study Chin et al [71], ANOVA and Pearson *χ^2^* test were used to analyze the age, education, and gender of the 3 cognitive groups. They mentioned that the results were significantly affected by age, gender, and years of education (especially in normal participants). Although Noguchi-Shinohara et al [73] applied the Kruskal-Wallis test and *χ^2^* test to compare age groups among cognitive conditions, they did not observe any significant differences between gender, age, and education. To summarize, diverse personal traits have been recognized in the existing literature as relevant factors that affect cognitive test performance. Some of the reviewed studies assessed the effects of age, gender, and educational level, whereas none of them considered statistical comparisons of cultural factors. Therefore, further studies are needed to delve deeper into the effects of diverse personal traits on cognitive test performance.

#### Psychometric Parameters

The main psychometric parameters considered were the different validation processes and measurement parameters presented in this study. A comparative matrix was created for the 20 selected articles by considering the available data and the mentioned psychometric parameters. The data included the type of population under observation, sensitivity, specificity, criteria for discriminating and comparing types of participants, and possible correlations for assessing validity.

Concerning the psychometric validation process, Table S1 Multimedia Appendix 1 [42,44-47,49,57-76] displays the key parameters, including sensitivity, specificity, area under the curve, and correlation validity, with some traditional tests (on paper) such as the MoCA, MMSE, and CVLT. This table was the most difficult to prepare because some tests had nonhomogeneous validation parameters.

In addition, other comparison variables are as follows (columns of the table in Appendix 1):

1. N (normal) is the number of people diagnosed and categorized as a control group based on clinical criteria (or people who are normally healthy or without cognitive impairment).
2. MCI indicates the number of people diagnosed with MCI based on clinical criteria (such as traditional psychometric tests or interviews).
3. DM (dementia) denotes the number of people evaluated and diagnosed with a certain degree of dementia based on clinical criteria (such as traditional psychometric tests or interviews).
4. OT (other pathologies) is the number of people diagnosed with other pathologies, such as Parkinson disease, Alzheimer disease, or stroke.
5. CA-DISCR (capacity to discriminate) indicates the objective of the digital test, which is to assess the instruments’ capacity to discriminate and differentiate people to validate the tests, for example, normal versus dementia or normal versus MCI.
6. SENSI: sensitivity (the proportion of people with a disease to be diagnosed who will have a positive result) has already been explained.
7. SPEC: specificity (the proportion of people without a disease to be diagnosed who will have a negative result) has already been explained.
8. ROC area is the statistical method (based on sensitivity and specificity) to evaluate the ability of a test to discriminate between individuals with and without a disease.
9. COM-VAL (comparison and validation): the traditional test (or tests), such as MoCA, MMSE, or others, used in the validation process of the digital test are identified. The instrument proposed in the article is validated by comparing it with traditional tests through statistics of the correlation coefficient used (such as Pearson, linear regression, or others). A correlation is then established (for the validity of the construct).
10. VALID-CO (validity and correlation): the correlation coefficients indicate the relationship of the test with the various (traditional) measurement instruments and their criteria, as well as the relationship that exists between the test and the construct. Construct validity refers to the adequacy of inferences made from observations or measurements (often the test results). Specifically, it determines whether a test measures the intended construct. The validity of a test indicates the extent to which it accurately measures the theoretical construct it is designed to measure and whether it can be used effectively for its intended purpose. A test is considered valid if it accurately measures what it claims to measure.

Although numerous modalities or methodologies are available to evaluate a patient, some of these applications lack sufficient studies on validity, reliability, and precision. In addition, the population sample for these modalities may be very small or may simply lack studies to support their implementation. Three basic, but complex, statistical tables were created, and they aimed to compare the validity parameters and the correlation of the digital applications with their traditional equivalent tests. Heterogeneity among the methods was significant. Most of the studies performed previous tests to categorize the participants into groups (using tools such as the MMSE, MoCA, or other specialized instruments) and subsequently validated the results through a correlation analysis. However, some applications are evaluated according to specific tests (tasks) rather than groups of patients with different characteristics. Some applications evaluated their area under the curve on age ranges [73], whereas others assessed it based on groups categorized by pathologies (such as healthy, MCI, dementia, or other combinations) [58,61,62,67,71,73-75].

In summary, the most usual values for validity cases were as follows:

- Sensitivity ranged from 0.63 to 0.95. With the exception of 2 very extreme cases, the lowest error rate was observed with the Tower of London application [57] when comparing errors made among patients with MCI (0.379), whereas the highest error rate was observed with the University of California, San Francisco application [62] when comparing healthy patients to those with dementia (1.00). The applications with the highest rates of sensitivity were the computerized platforms BHA [70,75], as well as the digital version eSAGE [58].
- The specificity ranged from 0.54, as reported by the CogEvo computerized battery [67], to 1.00, as observed in the eSAGE application [58], when comparing healthy individuals to those with dementia. The highest average among the different comparisons corresponded to the TMT-A and TMT-B tests [72].
- The computerized cognitive platforms that presented a higher ROC curve were the University of California, San Francisco [62] and BHA [75] applications.

For the correlations, the most frequent cases of validation were observed with the traditional tests, specifically 10 with the MMSE and 8 with the MoCA. Some researchers validated their results using specialized tests such as the CVLT [62], Addenbrooke’s Cognitive Examination-Revised [64], Repeatable Battery for the Assessment of Neuropsychological Status [68], and other specific measures [69,75]. The cases with the highest correlations corresponded to the computerized assessment battery for cognition [73], eSAGE [58], BHA [75], Inbrain [71] and Cognivue [69] applications, with average values of 0.75, 0.77, 0.77, 0.85, and 0.86, respectively. It is noteworthy that in 4 articles, evaluations of the sensitivity and specificity of the results were not included [64,68,69,76].

## Discussion

### Principal Findings

In this work, the systematic review and subsequent analysis of 20 recent articles were performed with technological alternatives to determine “the state of the art” of cognitive assessment tests in digital format. Of the 20 articles selected from 2015 onward, 14 (70%) were from 2019 to 2021, which demonstrates the growing interest in developing research on digital cognitive evaluation and training, particularly for older adults.

The *Results* section includes a discussion of each of the 4 research questions. An analysis of previous literature reviews yielded 4 papers published from 2015 to 2021. Our work provides a more up-to-date state-of-the-art approach, focusing on a wider range of cognitive domains. In addition to considering computer tests and digital test batteries, we included some initial studies on emerging technologies, such as VR, video games, gamification, and AI.

Computerized tests (and tasks), similar to paper-and-pencil tests, were found to generally assess the same domains but with different modalities. The most commonly used type is test batteries, which are administered on computers, tablets, or web platforms. There were also a few cases involving daily-life activity simulators, digital games, VR, and machine learning (AI). The use of these new technologies is expected to increase in the near future. Digital tests present improvements in completion place and time, do not require a structured system, and are portable, allowing cognitive evaluations to be carried out in the comfort of one’s home or community centers with minimal intervention from a specialist professional.

In addition, a well-designed digitized cognitive test allows for the reduction of “learning bias” by using pools of tasks to avoid repetition. In addition, it has the potential to be adapted to various contexts and people with different characteristics, thereby improving personalization and accessibility. However, these characteristics require further investigation.

Regarding the characteristics provided by the different digital tests, this work extends past reviews by providing details about the instructions and how they are delivered to the patient, the environment or place where the test is performed, and the sequence of tasks that the participants must perform on each of the tests. In addition, to enhance comparability among tests covering similar cognitive domains, we included their sample size, the details of how many participants had MCI or dementia, and the psychometric parameters used for each test in their comparative tables.

With regard to test validation, there is usually sufficient correlation between digital tests and their traditional paper-and-pencil counterparts, which validates their effectiveness in most cases. However, in many studies, the sample size of adults was small, and the psychometric parameters used were too heterogeneous, making comparisons difficult. It is also necessary to conduct further studies that consider differences in education level, gender, ethnicity, and culture.

Finally, most studies assume that the older adults use technology. However, an older person may not be familiar with the use of computers, tablets, or smartphones and may even need assistance in some cases. Therefore, it is necessary to incorporate other factors in future studies on digital tests, such as the degree of technology acceptance (Senior Technology Acceptance Model) as well as the emotional aspects of the participants, such as motivation and effort, along with usability evaluations.

## Appendix

Multimedia Appendix 1 Measurement parameters of the psychometric validation process.

Multimedia Appendix 2 PRISMA Checklist.

## Abbreviations

AI: artificial intelligence
BHA: Brain Health Assessment
CVLT: California Verbal Learning Test
eSAGE: Self-Administered Gerocognitive Examination
MCI: mild cognitive impairment
MMSE: Mini-Mental State Examination
MoCA: Montreal Cognitive Assessment
PRISMA: Preferred Reporting Items for Systematic Reviews and Meta-Analyses
ROC: receiver operating characteristic
STM: short-term memory
TMT: Trail Making Test
VR: virtual reality
VSM: Virtual Supermarket
