# Supplementary material for: Digital Cognitive Assessment Tests for Older Adults: Systematic Literature Review
Source: JMIR Ment Health. 2023 Dec 8;10:e47487. doi: 10.2196/47487 (PMC10746978; doi:10.2196/47487)
Supplement: Multimedia Appendix 1 [file mental_v10i1e47487_app1.pdf]

APPENDIX 1

Measurement Parameters of the Psychometric Validation Process.

A brief synthesis will be given on how to evaluate the quality of psychometric instruments; and in addition, the measurement parameters that are normally used.

Reliability

Reliability is defined as the consistency and precision of a measure on psychometric instruments. Depending on the scores that the person obtains, there will be some percentage of error, since it will be the real value of the patient added to it a percentage of error, since it may happen that the patient presents himself to take the psychological tests with different motivation and this causes the scores to not be totally consistent [49].

There are different ways to determine the reliability of a test [44]: Test-Retest; Inter-rater reliability; Parallel shapes; and halves. The reliability indices that are often used in neuropsychology are test-re-test reliability and inter-rater reliability, the important thing is that the reliability is greater than or equal to 0.80 [44].

Validity

Validity refers to the ability of a test to measure what it is supposed to measure, so it should not be expressed in general, but rather for the particular use for which the psychometric instrument is intended to be used. Procedures for determining validity emphasize the relationships between test performance and the link between theory and psychological verification through empirical and experimental testing of the hypothesis [49].

There are different types of validity [49] [42, 44]. Various methods are used to estimate construct validity [44, 45]. All these methods are complementary to each other, therefore there is no single measure for construct validity [45].

Sensitivity

Sensitivity indicates the probability of correctly classifying an individual (ie, characterizes the ability of the test to detect disease in diseased subjects). Technically, the sensitivity of a test is defined as the proportion of people with the disease who will test positive [46]. The higher the sensitivity, the lower the error rate when there are changes or false positives [44].

Specificity

Specificity indicates the probability that a subject will have a negative result in the test (or else, it is the ability to detect healthy individuals). Technically, the specificity of a test is defined as the proportion of people without the disease who will have a negative result [46]. In general, the tests to confirm the diagnosis must be of high specificity, to avoid false positives [44].

ROC Curves

Analysis based on ROC curves (receiver operating characteristic curve) is a statistical method to determine the diagnostic accuracy of tests that use continuous scales; and represents the exposure of people to the instrument (test to be used). The area under the ROC curves is a good global indicator of the precision of a diagnostic test (it makes it possible to express the performance of a test by means of a simple number); and it consists of a graphical technique to assess the ability of a test to discriminate between those with disease and those without disease [47]. ROC curves allow visual analysis of trade-offs between the sensitivity and specificity of a test with respect to the various cut-off points that can be used. The curve is obtained by calculating the sensitivity and specificity of the test at each possible cut-off point and plotting the sensitivity against specificity 1. This area is always greater than or equal to 0.5. The range of values moves between 1 (perfect discrimination) and 0.5 (there are no differences in the distribution of the test values between the 2 groups).

TABLE S1 : Main Parameters of the Psychometric Validation Process.

n/a: not available; no data

N°	Application or Battery	N	MCI	DM	OT	CA-DISCR	SENSI	ESPEC	ROC AREA	COM-VAL	VALID-CO
1.	TOL [57]	140	29	0	111	Breaking off - DCL<br>Mistakes - DCL	0,655<br>0,379	0,586<br>0,862	0,63<br>0,61	MoCA–Breaking<br>MoCA-Mistakes	- 0,161 a - 0,447<br>- 0,171 a -0,346
2.	eSAGE [58]	21	24	21	0	(DCL+DM) - N<br>N - DCL<br>N – DM<br>MoCA	0,71<br>0,63<br>0,95<br>0,91	0,90<br>0,81<br>1,00<br>0,67	0,88<br>0,78<br>0,99<br>0,88	SAGE ----<br>MMSE ----<br>MoCA ----	---- 0,882 p<0,0001<br>---- 0,6711<br>---- 0,7577
3.	CoCoSc [59]	101	59	0	0	N / DCL	0,78	0,69	0,78	MoCA ----	---- 0,71 p<0,001
4.	Episodix Gamification (CVLT) [60]	8	3	5	0	Support vector machine (Episodix +CVG)	0.77	0.90	0.90	MMSE ----<br>CVLT ----	---- 0,06 - 0,92<br>---- -1 a 1
5.	SIMBAC (IADL) [61]	81	52	28	0	N v/s DCL<br>N v/s DM<br>DCL v/s DM	0,79<br>0,86<br>0,71	0,58<br>0,75<br>0,77	0,77<br>0,97<br>0,82	MMSE ----<br>MMSE ----<br>MMSE ----	---- 0,65<br>---- 0,83<br>---- 0,69
6.	UCSF Brain Health Assessment [62]	185	99	42	21	N v/s DM<br>N v/s DCL	1,00<br>0,84	0,85<br>0,85	0,99<br>0,94	CVLT-II ----<br>CVLT-II ----<br>Benson ----<br>Dig.Sym ----	---- 0,77 (short)<br>---- 0,48 (stand)<br>---- 0,54<br>---- 0,83
7.	BrainCheack Inc [63]	398	0	0	185	They separate by tests, not by type of patient	0,81<br>0,89	0,94<br>0,78	n/a	SLUMS ----<br>MMSE ----<br>MoCA -----	-------- 0,81<br>-------- 0,62<br>------- 0,77
8.	CGN_ICA [64]	270				Does not discriminate patients (assesses accuracy and speed)	n/a	n/a	n/a	MoCA ----<br>ACE –R ----<br>MMSE ----	---- 0,46 - 0,55<br>---- 0.60, p <10-6)<br>---- 0,33, p <0,01
9.	VSM [65]	52	37	0	0	DCL and N<br>MMSE	0,74<br>0,71	0,85<br>0,85	n/a	Battery (rho) ---<br>Several (5)	---- 0,209 a 0,616<br>Several (5)
10.	CompBased-CAT [66]	746	27	0	0	N vs DCL	0,81	0,77	0,85	MMSE ----	---- 0,24 -0,51
11.	CogEvo [67]	121	103	48	0	N - (DM + DCL)<br>N - DCL<br>N - DM	0,70<br>0,78<br>0,85	0,60<br>0,54<br>0,70	n/a	MMSE ----	---- 0,2973

12.	CANTAB [68]	23				Evaluate for each task	n/a	n/a	n/a	Series RBANS:<br>Global<br>Attent/WorkMem<br>Processing speed<br>Memory<br>Executive funct	---- 0,71 , p<0,01<br>---- 0,48 . p<0,05<br>---- 0,51 , p<0,05<br>---- 0,42 , p<0,10<br>---- 0,64 , p<0,01
13.	Cognivue [69]	120	173	108	0	Positive and negative % agreement	n/a	n/a	n/a	SLUMS ----<br>Don´t compare with MMSE o MoCA	---- 0,82 a 0,90 p<0,001
14.	BHA- CS [70]	451	289	110	0	N v/s DCL (english)<br><br>N v/s DCL (spanish)	0,88<br>0,83<br>0,95<br>0,81	0,80<br>0,85<br>0,71<br>0,85	0,85	MoCA ----	---- 0,75
15.	Inbrain CST [71]	480	42	32	26	N vs (DCL+DM)<br>N vs DCL<br>(N+DCL) vs OT	0,846<br>0,808<br>0,824	0,845<br>0,762<br>0,828	0,879<br>0,812<br>0,930	MMES ----	---- 0,852
16.	Trail Making Test y Bells Test [72]	22	49	12	0	mistakes TMT-A<br>mistakes TMT-B<br>Bells	0,857<br>0,722<br>----	0,995<br>0,984<br>----	n/a	TMT-A ----<br>TMT-B ----<br>Bell ----	---- 0,68 p<0,001<br>---- 0,78 p<0,001<br>---- 0,39 p<0,001
17.	C-ABC [73]	367	137	336	0	DM vs (DCL+N)<br>MCI v/s N	0,83<br>0,77	0,72<br>0,64	0,854<br>0,741	MMSE ----	---- 0,753 p <0,001;
18.	EC-Screen [74]	126	54	63	0	(DCL + DM) vs N<br>DM vs (DCL + N)	0,81<br>0,83	0,80<br>0,83	0,87<br>0,90	HK-MoCA ----	---- –0,67 p<0,001
19.	BHA [75]	53	46	47	0	N vs (DCL+DM)<br>N vs DCL	0,91<br>0,71	0,85<br>0,85	0,95<br>0,94	Traditional tests	--- - 0,68 a 0,85
20.	serious game (SG) (IADL) [76]	23	23	25	20	Several cases N vs. (DCL+DM+OT)<br>Precision<br>Time<br>Distance<br>Total	n/a	n/a	0,975<br>0,937<br>0,237<br>0,982	Total:<br>MMSE -----<br>MoCA -----	----- 0,37 p<0,01<br>----- 0,64 p<0,01
