# Supplementary material for: Digital Cognitive Assessment Tests for Older Adults: Systematic Literature Review
Source: JMIR Ment Health. 2023 Dec 8;10:e47487. doi: 10.2196/47487 (PMC10746978; doi:10.2196/47487)
Supplement: Multimedia Appendix 2 [file mental_v10i1e47487_app2.pdf]

PRISMA 2020 Checklist

Section and Topic	Item #	Checklist item	Location where item is reported
**TITLE**			
Title	1	Identify the report as a systematic review.<br>*Digital Cognitive Assessment Tests for Older Adults: Systematic Review of the Literature*	√
**ABSTRACT**			
Abstract	2	See the PRISMA 2020 for Abstracts checklist.<br>*According to JMIR Editorial standards (Background, Objective, Methods, Results, Conclusions).*	√
**INTRODUCTION**			
Rationale	3	Describe the rationale for the review in the context of existing knowledge.<br>*In the Background of the Abstract, and in the final part of the Introduction.*	√
Objectives	4	Provide an explicit statement of the objective(s) or question(s) the review addresses.<br>*The objective is in the Abstract, and the research questions are at the beginning of the Methods (Objectives and Research Questions).*	√
**METHODS**			
Eligibility criteria	5	Specify the inclusion and exclusion criteria for the review and how studies were grouped for the syntheses.<br>*They are specified, and are included in the "Eligibility Criteria" paragraph.*	√
Information sources	6	Specify all databases, registers, websites, organisations, reference lists and other sources searched or consulted to identify studies. Specify the date when each source was last searched or consulted.<br>*The databases consulted were: Web of Science, PubMed and Scopus. The search work on the sources began during the month of March 2021.*	√
Search strategy	7	Present the full search strategies for all databases, registers and websites, including any filters and limits used.<br>*The search strategies and expressions are explained in the "Eligibility Criteria" paragraph.*	√
Selection process	8	Specify the methods used to decide whether a study met the inclusion criteria of the review, including how many reviewers screened each record and each report retrieved, whether they worked independently, and if applicable, details of automation tools used in the process.<br>*There were four reviewers: the two authors, and two student assistants. Everyone worked independently, and no automation tools were used in the process.*	√
Data collection process	9	Specify the methods used to collect data from reports, including how many reviewers collected data from each report, whether they worked independently, any processes for obtaining or confirming data from study investigators, and if applicable, details of automation tools used in the process.<br>*There were four reviewers: the two authors, and two student assistants. Everyone worked independently, and no automation tools were used in the process.*	√
Data items	10a	List and define all outcomes for which data were sought. Specify whether all results that were compatible with each outcome domain in each study were sought (e.g. for all measures, time points, analyses), and if not, the methods used to decide which results to collect.<br>*The outcomes for which data were sought are specified in the PRISMA diagram in Figure 3, and their explanation in the "Data Collection" paragraph.*	√
	10b	List and define all other variables for which data were sought (e.g. participant and intervention characteristics, funding sources). Describe any assumptions made about any missing or unclear information.<br>*The other variables for which data were sought are specified in the "Methods" Chapter, and were based on the research questions.*	√

PRISMA 2020 Checklist

Section and Topic	Item #	Checklist item	Location where item is reported
Study risk of bias assessment	11	Specify the methods used to assess risk of bias in the included studies, including details of the tool(s) used, how many reviewers assessed each study and whether they worked independently, and if applicable, details of automation tools used in the process.<br>*There were four reviewers: the two authors and two student assistants. Everyone worked independently, the risk of bias assessment was based on the experience of each reviewer, and no automation tools were used in the process.*	√
Effect measures	12	Specify for each outcome the effect measure(s) (e.g. risk ratio, mean difference) used in the synthesis or presentation of results.<br>*For the synthesis and presentation of the results, several comparative tables were considered that are included in the text of the Review. Even a Table in Multimedia Appendix 1.*	√
Synthesis methods	13a	Describe the processes used to decide which studies were eligible for each synthesis (e.g. tabulating the study intervention characteristics and comparing against the planned groups for each synthesis (item #5)).<br>*The decision of which studies were eligible for each synthesis is described in the Methods Chapter paragraphs, and is based on the eligibility criteria, and how they satisfied the research questions.*	√
	13b	Describe any methods required to prepare the data for presentation or synthesis, such as handling of missing summary statistics, or data conversions.<br>*For the synthesis and presentation of the data, comparative tables were used, using summary statistics of the studies, and how they satisfied the research questions.*	√
	13c	Describe any methods used to tabulate or visually display results of individual studies and syntheses.<br>*To tabulate or visually display the results of studies and individual syntheses, electronic spreadsheets were used. And several tables are included in the Review.*	√
	13d	Describe any methods used to synthesize results and provide a rationale for the choice(s). If meta-analysis was performed, describe the model(s), method(s) to identify the presence and extent of statistical heterogeneity, and software package(s) used.<br>*To synthesize the results and justify the choice, eligibility and inclusion criteria were used, according to the research questions and the objective of the Review. A meta-analysis was not performed as it was not included as an objective in the Review.*	√
	13e	Describe any methods used to explore possible causes of heterogeneity among study results (e.g. subgroup analysis, meta-regression).<br>*To explore possible causes of heterogeneity between the results of the studies, the eligibility and inclusion criteria were used, according to the research questions, and the objective of the Review.*	√
	13f	Describe any sensitivity analyses conducted to assess robustness of the synthesized results.<br>*To evaluate the robustness of the synthesized results of the studies, sensitivity and specificity analyzes were done, which are explained in Multimedia Appendix 1.*	√
Reporting bias assessment	14	Describe any methods used to assess risk of bias due to missing results in a synthesis (arising from reporting biases).<br>*To assess the risk of bias due to possible missing results, we involved four reviewers: the two authors and two student assistants. Everyone worked methodically and independently, and the risk of bias assessment was based on the experience of each reviewer.*	√
Certainty assessment	15	Describe any methods used to assess certainty (or confidence) in the body of evidence for an outcome.<br>*To evaluate the certainty or confidence in the body of evidence for an outcome, four reviewers participated: the two authors and two student assistants. Everyone worked methodically and independently, and the assessment of certainty was based on the experience of each reviewer .*	√

PRISMA 2020 Checklist

Section and Topic	Item #	Checklist item	Location where item is reported
**RESULTS**			
Study selection	16a	Describe the results of the search and selection process, from the number of records identified in the search to the number of studies included in the review, ideally using a flow diagram.<br>*The results of the search and selection process are described in the diagram in Figure 3.*	√
	16b	Cite studies that might appear to meet the inclusion criteria, but which were excluded, and explain why they were excluded.<br>*The studies that were excluded from the selection and why, are shown in the diagram in Figure 3.*	√
Study characteristics	17	Cite each included study and present its characteristics.<br>*As explained in the Review, the included studies were 20, and their main characteristics are shown in Tables 1, 5 and 9 of the Review.*	√
Risk of bias in studies	18	Present assessments of risk of bias for each included study.<br>*Of the 20 studies included, and their possible risk of bias, their evaluation is explained in the paragraphs of the Results and Discussion Chapters*.	√
Results of individual studies	19	For all outcomes, present, for each study: (a) summary statistics for each group (where appropriate) and (b) an effect estimate and its precision (e.g. confidence/credible interval), ideally using structured tables or plots.<br>*For the results of the studies, a set of statistics and characteristics of all the digital cognitive tests analyzed are presented, using tables structured in the Review. In particular, Tables 5, 7, 8 and 9.*	√
Results of syntheses	20a	For each synthesis, briefly summarise the characteristics and risk of bias among contributing studies.<br>*The characteristics of the contributing studies are summarized in all Tables (1 to 9).*	√
	20b	Present results of all statistical syntheses conducted. If meta-analysis was done, present for each the summary estimate and its precision (e.g. confidence/credible interval) and measures of statistical heterogeneity. If comparing groups, describe the direction of the effect.<br>*Los resultados de las síntesis estadísticas realizadas, se presentan en las Tablas (1 a 9). A meta-analysis was not performed as it was not included as an objective in the Review.*	√
	20c	Present results of all investigations of possible causes of heterogeneity among study results.<br>*The results of all the investigations on possible causes of heterogeneity between the results of the studies can be seen in Tables 1, 4, 5 and 9 of the Review.*	√
	20d	Present results of all sensitivity analyses conducted to assess the robustness of the synthesized results.<br>*To evaluate the robustness of the synthesized results of the studies, sensitivity and specificity analyzes were done, which are explained in Multimedia Appendix 1.*	√
Reporting biases	21	Present assessments of risk of bias due to missing results (arising from reporting biases) for each synthesis assessed.<br>*To assess the risk of bias due to possible missing results, we involved four reviewers: the two authors and two student assistants. Everyone worked methodically and independently, and the risk of bias assessment was based on the experience of each reviewer. Assessments of risk of bias due to possible missing results are discussed in the Results and Discussion Chapters.*	√
Certainty of evidence	22	Present assessments of certainty (or confidence) in the body of evidence for each outcome assessed.<br>*To evaluate the certainty or confidence in the body of evidence for an outcome, four reviewers participated: the two authors and two student assistants. Everyone worked methodically and independently, and the assessment of certainty was based on the experience of each reviewer.*	√

PRISMA 2020 Checklist

Section and Topic	Item #	Checklist item	Location where item is reported
**DISCUSSION**			
Discussion	23a	Provide a general interpretation of the results in the context of other evidence.<br>*A general (and in some cases detailed) interpretation of the results in the context of other evidence is provided. In the Principal Findings paragraph.*	√
	23b	Discuss any limitations of the evidence included in the review.<br>*The possible limitations of the evidence included in the Review are analyzed and discussed.*	√
	23c	Discuss any limitations of the review processes used.<br>*The possible limitations of the review processes used are analyzed and discussed.*	√
	23d	Discuss implications of the results for practice, policy, and future research.<br>*The implications of the results obtained from the Review for the practice of professionals, and the need to incorporate other factors in future research on digital cognitive tests, are analyzed and discussed.*	√
**OTHER INFORMATION**			
Registration and protocol	24a	Provide registration information for the review, including register name and registration number, or state that the review was not registered.<br>*The review was registered for the standards of the JMIR Publishing House, and corresponds to registration # 47487, of the Mental Health publication (JMIR Ment Health 2023;10:e47487).*	√
	24b	Indicate where the review protocol can be accessed, or state that a protocol was not prepared.<br>*The review was carried out under the standards of the JMIR Editorial, and following the steps detailed in the Methods Chapter of the Review (no special protocol was prepared).*	√
	24c	Describe and explain any amendments to information provided at registration or in the protocol.<br>*There are no modifications to the information provided.*	√
Support	25	Describe sources of financial or non-financial support for the review, and the role of the funders or sponsors in the review.<br>*There were no external sources of financial support for the review. It was financed only with the support of the authors.*	√
Competing interests	26	Declare any competing interests of review authors.<br>*Both authors of the Review are academics and University researchers.*	√
Availability of data, code and other materials	27	Report which of the following are publicly available and where they can be found: template data collection forms; data extracted from included studies; data used for all analyses; analytic code; any other materials used in the review.<br>*The information that is publicly available corresponds to the numerous References cited in the Review. Other background information can be obtained by contacting the authors.*	√

From: Page MJ, McKenzie JE, Bossuyt PM, Boutron I, Hoffmann TC, Mulrow CD, et al. The PRISMA 2020 statement: an updated guideline for reporting systematic reviews. BMJ 2021;372:n71. doi: 10.1136/bmj.n71

For more information, visit: http://www.prisma-statement.org/
